# CluePedia Cytoscape plugin: pathway insights using integrated experimental and *in silico* data

**DOI:** 10.1093/bioinformatics/btt019

**Published:** 2013-01-16

**Authors:** Gabriela Bindea, Jérôme Galon, Bernhard Mlecnik

**Affiliations:** ^1^INSERM, Laboratory of Integrative Cancer Immunology, 75006 Paris, ^2^Cordeliers Research Center, Université Paris Descartes, 75006 Paris and ^3^Cordeliers Research Center, Université Pierre et Marie Curie Paris 6, Cordeliers Research Center, 75005 Paris, France

## Abstract

**Summary**: The CluePedia Cytoscape plugin is a search tool for new markers potentially associated to pathways. CluePedia calculates linear and non-linear statistical dependencies from experimental data. Genes, proteins and miRNAs can be connected based on *in silico* and/or experimental information and integrated into a ClueGO network of terms/pathways. Interrelations within each pathway can be investigated, and new potential associations may be revealed through gene/protein/miRNA enrichments. A pathway-like visualization can be created using the Cerebral plugin layout. Combining all these features is essential for data interpretation and the generation of new hypotheses. The CluePedia Cytoscape plugin is user-friendly and has an expressive and intuitive visualization.

**Availability**: http://www.ici.upmc.fr/cluepedia/ and via the Cytoscape plugin manager. The user manual is available at the CluePedia website.

**Contact**: bernhard.mlecnik@crc.jussieu.fr or jerome.galon@crc.jussieu.fr

**Supplementary information:** Supplementary data are available at *Bioinformatics* online.

## 1 INTRODUCTION

Integrating heterogeneous expression data and functional network information is essential for understanding cellular processes and their dynamics. Within the versatile framework of Cytoscape (Saito *et al.*, 2012; Shannon *et al.*, 2003), tools for ontology analysis (Bindea *et al.*, 2009) and gene function predictions (Montojo *et al.*, 2010) were developed. CluePedia provides a comprehensive view on a pathway or process by investigating experimental and *in silico* data from different perspectives: gene interrelations, miRNAs regulatory aspects, protein–protein interactions, as well as the functional context, in conjunction with ClueGO (Bindea *et al.*, 2009).

## 2 METHODS AND IMPLEMENTATION

### 2.1 Data sources, import and update

The user can analyze his/her own experimental data and directly compare and enrich it with publicly available information from STRING (Szklarczyk *et al.*, 2011), IntAct (Kerrien *et al.*, 2012), MiMI (Tarcea *et al.*, 2009), miRBase (Kozomara and Griffiths-Jones, 2011) and miRecords (Xiao *et al.*, 2009). The pathway analysis is based on GO (Ashburner *et al.*, 2000), KEGG (Kanehisa *et al.*, 2002), Reactome (Croft *et al.*, 2011) and other resources (Supplementary Material).

Sets of identifiers of interest can be directly uploaded in text format, pasted in a text field or interactively derived from gene networks. The plugin automatically recognizes a variety of identifiers for genes, proteins or miRNAs that can be updated with the latest NCBI information.

CluePedia comes with human and mouse interaction data. New pre-compiled files as well as data for >20 other organisms can be automatically downloaded after the installation. The plugin is easily extendable for additional organisms and identifiers in a plugin-like manner.

### 2.2 Pathway interactions from custom and *in silico* data

CluePedia calculates statistical dependencies (correlation) for markers of interest from experimental data. Four tests for investigating linear and non-linear dependencies between variables are implemented: Pearson correlation, Spearman’s rank, Distance correlation (Szekely and Rizzo, 2009) and the recently described Maximal Information Coefficient (MIC) (Reshef *et al.*, 2011). These tests can be applied simultaneously or individually to analyze whole-input file (e.g. Affymetrix datasets), selected markers versus the entire dataset or among selected markers only. The resulting file is added to CluePedia as an additional resource for further analysis.

Experimental data can be normalized and visualized next to the nodes using adjustable thresholds. Filter methods based on the expression level, standard deviation and the number of missing values are available. Another feature allows the extraction of a subset of expression data corresponding to a pathway or term, from a dataset into a new file.

### 2.3 Enrichment of pathways

CluePedia visualizes custom correlation weights as well as known interaction and miRNA-binding scores as edges on the network.

The network can be enriched with markers with the highest interaction score for all or each of the selected nodes. Furthermore, the network can be enriched with hub markers that have the highest connectivity with all selected nodes. Different edge score types can be used together for the enrichment, and the network will be updated with the specified number of top scored interactions meeting all threshold criteria. Customizable filters allow the visualization of highest common/specific interaction scores within the data sources selected. The user can easily modify the action type displayed (e.g. activation, inhibition), as well as the color of nodes and edges.

Importantly, this enrichment can be used within ClueGO networks of pathways. Newly enriched genes already known to be associated with a pathway will be automatically linked to it. Another original feature of CluePedia expands ClueGO terms into nested networks. Like this, a pathway can be investigated in detail to reveal how known gene interrelations are modulated within the experimental context used, and which could be the newly associated genes/miRNAs.

CluePedia automatically extracts the cellular location of markers from GO terms and maps it on pre-defined cellular compartments. Based on this information, a pathway-like view can be created using the Cerebral plugin layout (Barsky *et al.*, 2007). If for some markers no cellular location is found, they will be placed into a ‘no annotation found’ layer. The provided cellular compartments can be easily modified and extended by the user. The network and the pathway views can easily be switched.

## 3 CASE STUDY

**Fig. 1.**
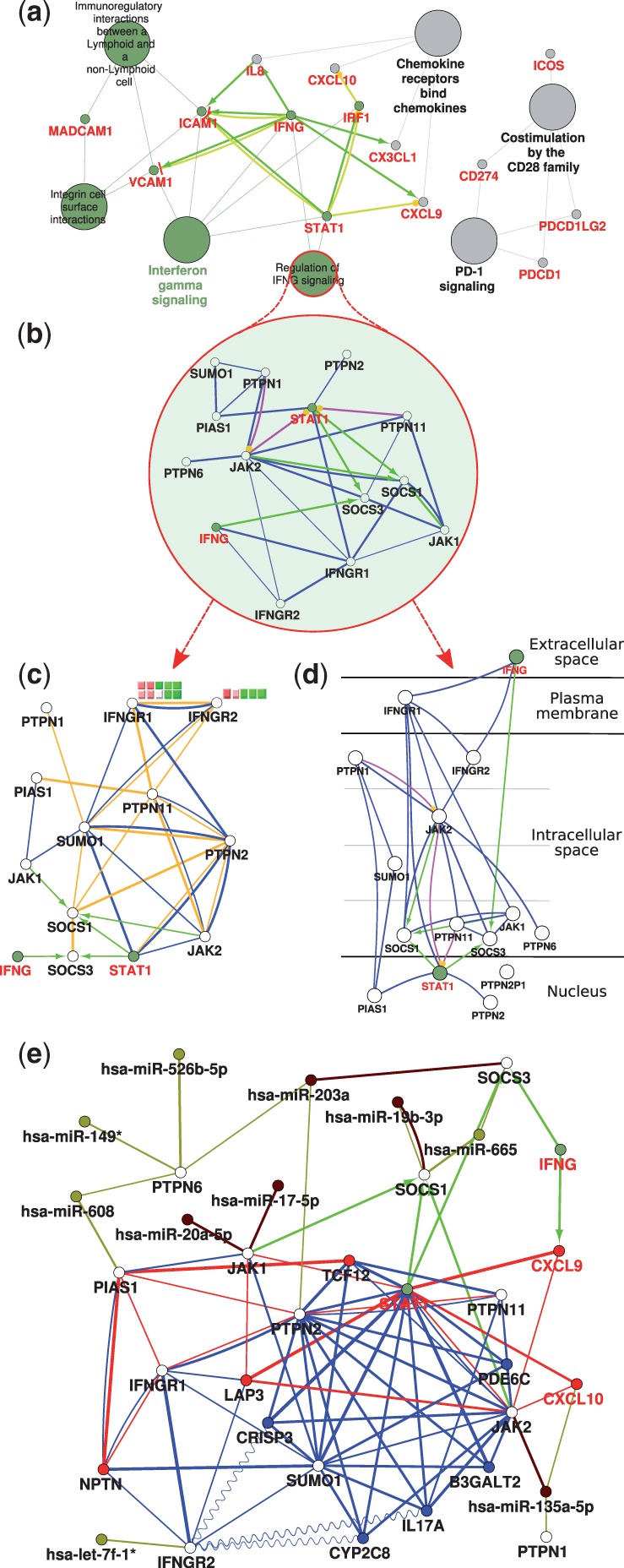
CluePedia analysis example using expression data from human normal colon mucosa and colorectal tumors and in silico information. (**a**) Functionally grouped network with pathways and genes. Terms are linked based on κ score (≥0.3). Edges show known activation (green), expression (yellow), post-translational modification (pink) and binding (blue). The edge thickness is scaled between the minimum and maximum scores shown. Terms and their associated genes share the color. ‘Regulation of IFNG signaling’ pathway is investigated in a subnetwork. (**b**) Genes not included in the initial selection are colored in white. Known interactions are shown. (**c**) Gene interrelations in normal samples are shown as Pearson correlation (blue) and MIC (orange), all values >0.7. Normalized expression data from five normal colon samples are shown as node label for IFNGR1 and IFNGR2. All spots corresponding to a gene are shown. (**d**) Pathway-like view of the network showing cellular locations. (**e**) ‘Regulation of IFNG signaling’ after enrichment steps. Five strongest correlating genes (Pearson) in normal colon and tumors, as well as the top five validated and predicted miRNAs (prediction score) are shown in blue, red, brown and beige, respectively. Negative correlations are shown as sinusoidal lines

T helper 1-related genes (Mlecnik *et al.*, 2010) and their corresponding pathways are simultaneously visualized in a CluePedia network (Fig. 1a). The ‘Regulation of IFNG signaling’ pathway is investigated as a nested network. *In silico* interactions within the pathway (Fig. 1b) can be compared with linear and non-linear interrelations derived from expression data from normal colon and colon tumors [E-MTAB-57 (Ancona *et al.*, 2006), E-GEOD-37892, ArrayExpress (Parkinson *et al.*, 2011)]. Normalized gene expression data are visualized next to the node (Fig. 1c). The network can be switched to a pathway-like view (Fig. 1d), displaying the cellular location of genes. The pathway is enriched with correlating genes from normal and tumor data as well as with predicted miRNAs that could play a crucial role in modulating interferon gamma (IFNG) signaling regulation (Fig. 1e).

## 4 SUMMARY

CluePedia allows a fast comparison of known and experimentally derived interrelation information. New gene/miRNA associations specific for the experimental context can be uncovered through enrichments and integrated into ClueGO networks of pathways. With CluePedia, the user can create a custom encyclopedia of interrelation sets. CluePedia Cytoscape plugin is freely available at http://www.ici.upmc.fr/cluepedia/ and via the Cytoscape plugin manager.

*Funding:* INCa, Canceropole Ile de France, INSERM, MedImmune, Qatar National Research Fund (NPRP09-1174-3-291); European Commission 7FP (Geninca, 202230); LabEx Immuno-Oncology.

*Conflict of Interest*: none declared.

## Supplementary Material

Supplementary Data
