# Supplementary material for: CluePedia Cytoscape plugin: pathway insights using integrated experimental and in silico data
Source: Bioinformatics. 2013 Jan 16;29(5):661–3. doi: 10.1093/bioinformatics/btt019 (PMC3582273; doi:10.1093/bioinformatics/btt019)
Supplement: Supplementary Data [file supp_29_5_661__index.html]

CluePedia Cytoscape plugin: pathway insights using integrated experimental and in silico data — Supplementary Data 

# CluePedia Cytoscape plugin: pathway insights using integrated experimental and *in silico* data

## Supplementary Data

files

**Files in this Data Supplement:**

- Supplementary Data - pdf file
