# Supplementary material for: CluePedia Cytoscape plugin: pathway insights using integrated experimental and in silico data
Source: Bioinformatics. 2013 Jan 16;29(5):661–3. doi: 10.1093/bioinformatics/btt019 (PMC3582273; doi:10.1093/bioinformatics/btt019)
Supplement: Supplementary Data [file supp_btt019_BIOINFO-btt019_supplementaryInformation.pdf]

CluePedia

Documentation

Gabriela Bindea, Bernhard Mlecnik
Laboratory of Integrative Cancer Immunology
INSERM U872 Cordeliers Research Center
Paris, France

Contents

Summary 2

Installation 4

Documentation 5

CluePedia Panels 5

CluePedia functionality 7

Gene/miRNA expression visualization/saving options 7

Use Cerebral layout 8

Expression data visualization 9

Close expression dataset 13

Expression data extraction 13

Save all selected genes 14

Gene/Interaction Enrichment Options 14

Enrich (Find new associations for an initial set of markers) 15

Add (Include a new set of markers to the current selection) 17

Remove (Discard enriched/added markers) 17

Network update (Refresh the network view with selected options) 17

Interactions 17

Known interactions 18

Interactions from custom data 18

Implemented statistical tests 20

Interactions visualization (edges) 22

Edge scores 22

Edge actions 23

Undirected edge filters . . . 23
Directed edge filters . . . 25
Download interactions for other organisms/Update interactions . . . 26
ClueGO+CluePedia functionality . . . 26
Expand pathways in nested networks . . . 26
Extract from pathways genes interracting with genes from initial list . . . 27
How to... . . . 33
Visualize known interactions . . . 33
Calculate interactions between markers using experimental data . . . 34
Make gene/miRNA enrichment . . . 36
Find functions that could be affected by miRNAs . . . 38
Enrich a network showing two lists of genes/miRNAs . . . 40
Switch the network and pathway-like view . . . 40
Upload new available example and ontology files or new organisms . . . 41
See node and edge attributes . . . 42
Data format . . . 42
Publicly available expression data . . . 43
Organism and ID types . . . 44
Update Ontologies and Interaction data . . . 44
Ontologies and *in silico* data sources . . . 46
Bibliography . . . 49

Summary

CluePedia provides insights into pathways by integrating experimental and in silico information.

CluePedia extends ClueGO [1] functionality down to genes and miRNAs. If ClueGO reveals interrelations of terms and functional groups in biological networks, CluePedia gives the posibility to enrich those networks with known and experimental data.

CluePedia calculates statistical dependencies (correlation) for markers of interest from experimental data. Four tests investigating linear and nonlinear dependencies between variables are implemented: Pearson correlation, Spearman's rank, Distance correlation ([2]) and Maximal Information Coefficient (MIC) ([3]). The resulting file is added to CluePedia as an additional resource for further analysis.

Experimental data can be normalized and visualized between adjustable thresholds on network's nodes as a label. Relevant signals with a certain expression level, standard deviation and without missing values can be selected. Another feature allows the extraction of expression corresponding to selected markers, e.g. genes associated with a pathway, from a dataset into a new file.

One major advantage is the possibility to investigate in detail a pathway by combining known [4, 5] and new, experimental derived information, about genes/proteins involved. More, miRNAs [6, 7] that could influence the expression of the genes can be as well visualized together with the expression data. The location of the genes/proteins [8, 9] within cellular compartments can be automatically displayed, by the implementation of Cerebral [10] layout within CluePedia.

CluePedia provides an ID index file (updatable, extendable) that stores entrez gene ids and symbols and allows fast analysis.

Both plugins, CluePedia and ClueGO, take advantage of Cytoscape's [11] versatile visualization framework.

Table 1 summarizes CluePedia features in comparison with ClueGO.

		ClueGO v.1.3	ClueGO v.1.5	CluePedia v.1.0
I.	Requirements	512MB RAM (1024 MB RAM recom.). Hard disk: >100MB free. Java 1.5+ (1.6 recom.). Cytoscape 2.6.+, -Xmx1024m	512MB RAM (1024 MB RAM recom.). Hard disk: >100MB free. Java 1.6. Cytoscape 2.8.3, -Xmx1024m	2048MB RAM (4096 MB RAM recom.). Java 1.6. Cytoscape 2.8.3, -Xmx4096m
	Dependencies	Cytoscape	Cytoscape	Cytoscape. ClueGO v1.5.
II.	Organisms	15 organisms	23 organisms	17 organisms
	Ids	Gene, protein ids	Automatic detection of id types	Automatic detection of id types
III.	Analysis Type	Functions. Single. Comparison	Functions. Single. Comparison	Genes. Single. Comparison
	Functional analysis	YES	YES	YES, in combination with ClueGO
	Analysis from custom data	NO	NO	Extract corresponding data for network genes from external files. Expression and Correlation modules. Enrich and Add genes/miRNAs
	Stats	Fisher Exact Test. Reference: Selected Ontologies	Fisher Exact Test. Reference: Selected Ontologies. Predefined IDs. Custom Set	MIC: Maximal Information Coefficient[3], Pearson, Spearman and Distance[2] correlation,
IV.	Visualization Types	Network of terms. Charts. Tables	Network of terms. Charts. Tables	List of found genes with synonym ids. Networks of: genes, genes and terms, genes and miRNAs, genes with expression data. Nested networks for a pathway/GO term. Pathway view. Edge actions with directed links. Tables
	Edges	Kappa Score	Kappa Score	Kappa Score. Scored edges. Custom correlation calculated from expression data
	Edge Scores[5]	NO	NO	Coexpression, combined score, co-occurence, database, experimental, fusion, neighorhood, textmining
	Edge Actions[5]	NO	NO	Activation, binding, expression, ptmod
	Layout	Organic	Organic	Organic. Cerebral
	Set Network Specificity	YES. GO level. #,% genes/term. Kappa score level. # of terms/group	YES. GO level. #,% genes/term. Kappa score level. # of terms/group	YES. Edges scores/actions
V.	Ontologies/ Data sources	GO, GOSlim. KEGG. Reactome	GO[8], GOSlim. KEGG[9]. Reactome[14]. PO Plants[15]. CORUM[17] SGD[17], Cyc2008[18], MetaCyc[19]	Interaction data[5]. miRNA binding site predictions[6,7] Experimental data
	Specific GO	NO	YES. Created from a root GO ID	NO
VI.	Automatic Update	GO. KEGG	GO. KEGG. Reactome. Annotation, Custom GO, GOSlim, PO	Interaction data and miRNA predictions from ICI site
	Combinations	GOlorize	GOlorize	GOlorize. Cerebral
	Memory bar	NO	YES	YES

Installation

System Requirements:

- Windows, Linux, Unix or MacOS operating system.
- 2048MB RAM needed, 4096 MB RAM recommended. Hard disk with at least 1Gb free.
- Java 1.5+ needed, Java 1.6 recommended.
- Cytoscape 2.8.+ installed, with -Xmx4096m option in the start up file (.sh/.path).
- ClueGO plugin 1.5.+ installed.
- *Optional:* Cerebral plugin installed.

CluePedia is a Cytoscape plugin and works together with ClueGO plugin, thus it is necessary to copy both, CluePediaPlugin.jar and ClueGOPlugin.jar in the plugins folder of Cytoscape.
CluePedia: http://www.ici.upmc.fr/cluepedia/cluepediaDownload.shtml.
ClueGO: http://www.ici.upmc.fr/cluego/cluegoDownload.shtml

For a pathway-like view of functional terms, Cerebral plugin jars (cerebral2.8.2.jar, prefuse.jar) should be downloaded in the plugins folder of Cytoscape.
Cerebral: http://pathogenomics.ca/cerebral/download.html

CluePedia and ClueGO source files will be stored in the .cluegoplugin folder. This folder is created in the user home folder at the first startup. If this folder is removed or the content damaged, it will be recreated automatically at the next startup, with the initial configuration.

CluePedia will take at the first run several minutes to initialize.

For miRNA analysis, please download intially available miRNA annotations (See Upload new available example and ontology files or new organisms).

Documentation

After starting Cytoscape, CluePedia and ClueGO can be found under the Plugins menu (Fig 1).

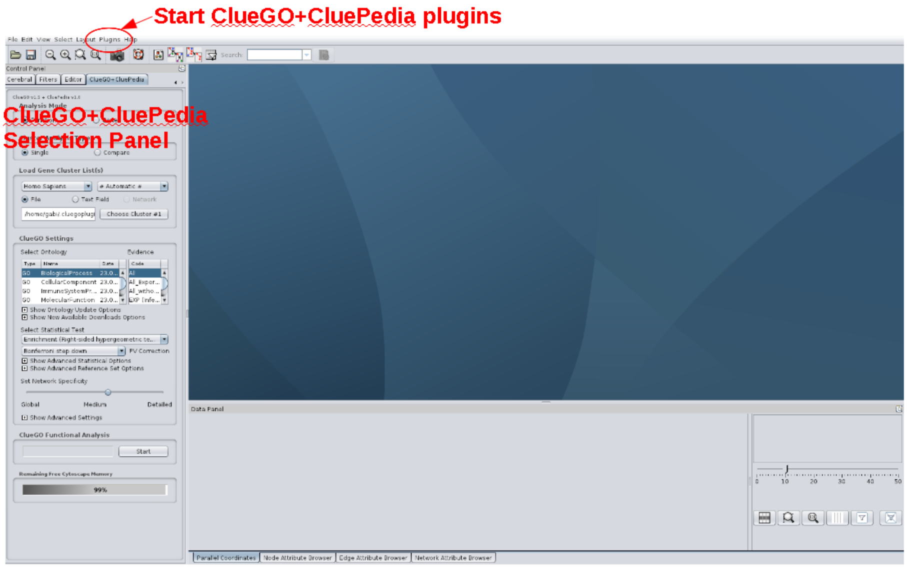

Figure 1: *Start ClueGO+CluePedia Plugins*

CluePedia (Fig 2) can be accessed by selecting Gene analysis mode:

- The two analysis modes: Functional analysis (ClueGO) and Gene based analysis (CluePedia); can be used in combination.
- Single and compared cluster analysis type are available for both analysis modes.
- The ID type is automatically identified for supported organisms.
- The identifies can be uploaded from File, Text Field or from an opened Network.
- Start. Genes are mapped against the ID index file. The extracted info is displayed in Gene Info Panel. Genes are shown as nodes of the network.
- Cytoscape Free Memory Bar estimates the remaining memory for Cytoscape

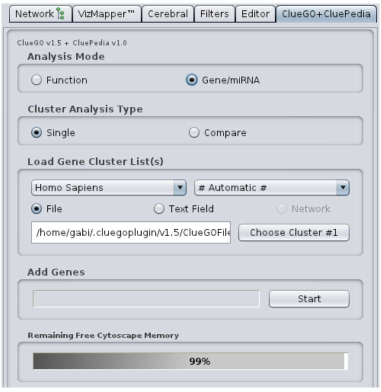

Figure 2: *CluePedia-ClueGO selection Panel, Gene/miRNA based analysis*

CluePedia Panels

After mapping a set of genes, a Gene network, a Gene info and a CluePedia panels are created (Fig 3).

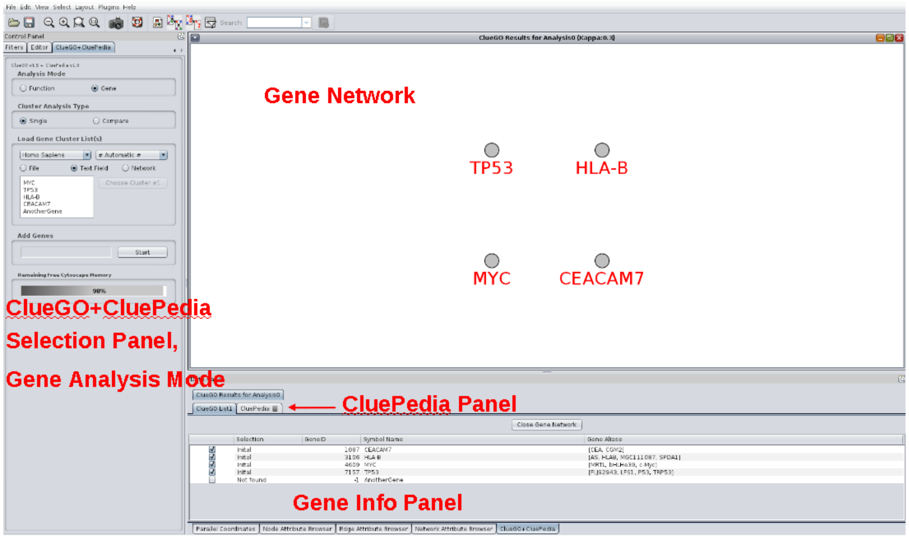

Figure 3: *CluePedia Panels*

Gene Info Panel:

- Shows EntrezGeneID, symbol and gene aliases for found genes.
- Not found genes are listed as well.

- Gene information can be directly saved (copy/paste) or using Cytoscape features, from the Node attribute table.
- Close Gene Network. Closes the network and associated tables.

CluePedia Panel:

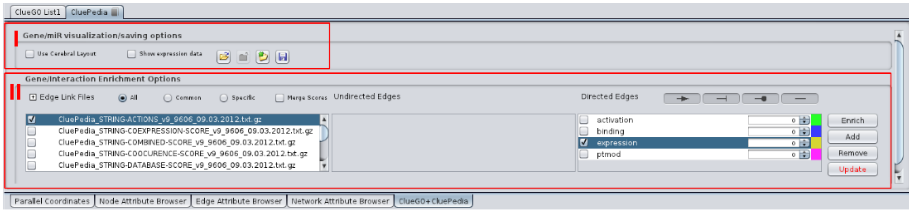

Figure 4: *CluePedia Panel*

CluePedia functionality

CluePedia panel (Fig 4) contains visualization and statistical options.

I. Gene/miRNA expression visualization/saving options.

   This function includes the cellular location layout and the experimental data selection, extraction, normalization and visualization.

II. Gene/Interaction Enrichment Options.

   Includes files with interaction and prediction scores.

   CluePedia built-in statistical tests for calculating correlation weights from custom data.

   Scores and directed and undirected edges refinement options.

   Nodes and edges enrichment, adding, removing and update features.

I. Gene/miRNA expression visualization/saving options

Provides the following options (from left to right):

1. Use Cerebral layout

2. Show expression data

3. Open expression dataset

4. Close expression dataset

5. Extract selected genes from dataset

6. Save all selected genes

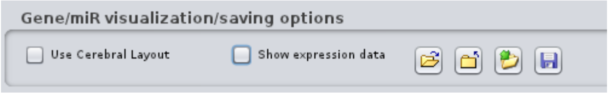

Figure 5: *Expression visualization options*

Use Cerebral layout

The cellular localization of the genes is automatically extracted during the mapping of the genes. The source of annotation is Cellular Component, Gene Ontology (GO) [8].

6 cellular compartments were defined: 1.Extracellular, 2.Plasma membrane, 3.Intra Cellular, 4.Nuclear Membrane, 5.Nucleus, 6.Transcription Factor Complex.

Each of those compartments summarizes a set of GO terms related to the cellular area described. For instance, the compartment Nucleus contains: nuclear part (GO:0044428), nucleolus (GO:0005730), nucleolar part (GO:0044452). The numbers show the order in which the layer are drawn.

Following the provided pattern, the user can change the source of annotation, include other GO terms in the existing cellular compartments or add new categories and complexes in .properties file.

If a gene is located in several compartments, those levels will be displayed together as a new category.

The visualization of the cellular location is done using the Cerebral layout (Fig 6).

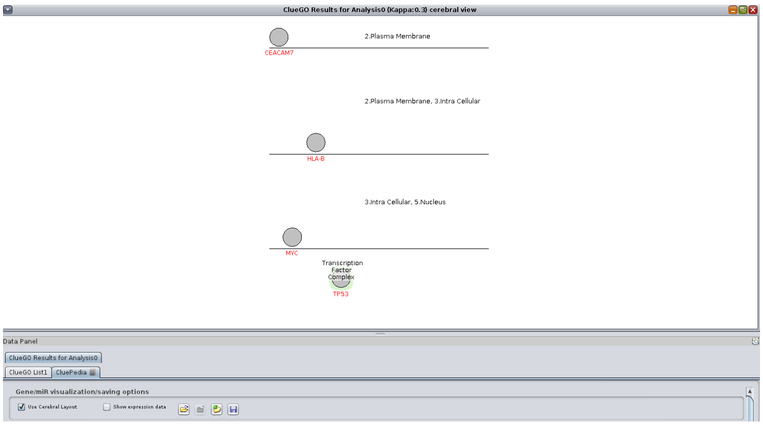

Figure 6: *Found Genes in cellular compartments*

Improvements starting with CluePedia v1.7

The cellular compartments were better defined by increasing the number of associated GO terms. Like this, a higher number of markers are mapped.

In comparison to previous CluePedia versions, in which the not mapped markers were randomly associated with a compartment, now they are placed in a ”annotation definition not found” group. This group is placed at the exterior of the other compartments. The user can verify the not mapped markers and add the eventually missing GO terms in the .properties file.

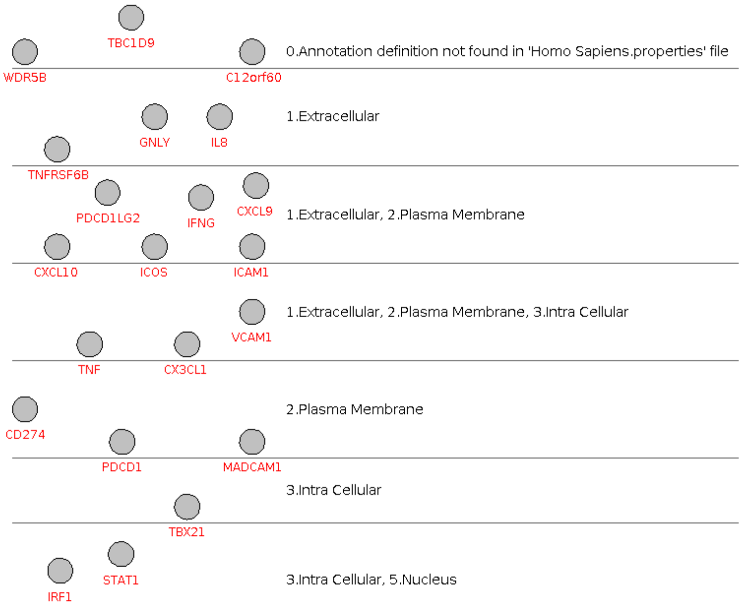

Figure 7: *Found Genes in cellular compartments, CluePedia v1.7+*

Expression data visualization

Expression data can be visualized as a node label (Show expression data).

After opening the custom file with expression data, the number of found genes and experiments/time points is shown. Several visualization options are available for the user. If two or more filters are set, only genes passing all filters will be visualized (AND relationship). The filtering and the normalization are done with the complete dataset.

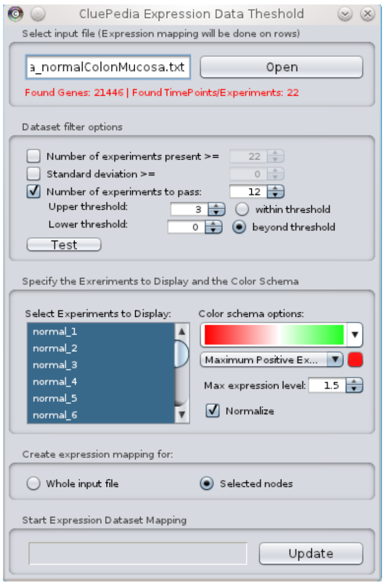

Figure 8: *Expression data visualization. Filters*

Filters allow for a fast investigation of the entire dataset. The user can discard genes with missing values, see which are the genes most changing in different experiments, or select genes with a certain level of expression.

- Number of experiments present >=

  Specifies the minimum number of experiments with data for a gene to be selected. Genes with more missing values than the threshold will be discarded. (Fig 9a). E.g. If the number of experiments present is set 6, 3 out of 5 genes will be selected.

- Standard deviation >=

  Selects the genes having the standard deviation bigger than the set threshold. Genes that change most over the experiments could be visualized.

- Number of experiments to pass:

  Selects genes with a specified expression level. E.g. If the expression of a gene ranges from -5 to 5 in a set of 6 experiments. The number of experiments to pass a threshold set to 3 is 4 (within threshold, -3 $<$ expression $<$ 3) and 2 (beyond threshold, expression $<$ -3, expression $>$ 3) (Fig 9b).

	Exp1	Exp2	Exp3	Exp4	Exp5	Exp6	
Gene1	5.82	5.89	6.17	4.32	5.25	5.82	selected
Gene2	5.23	NaN	NaN	NaN	4.86	0.00	not selected
Gene3	3.80	3.74	4.14	4.53	4.59	3.80	selected
Gene4	2.70	2.58	2.55	2.56	2.64	0.00	selected
Gene5	5.71	6.47	NaN	2.75	2.96	5.71	not selected

a)

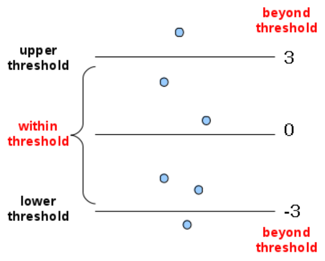

b)

Figure 9: *Expression filters example*

The user can select and visualize one or several experimental conditions.

Data can be normalized. The normalization is calculated as mean substraction divided by the standard deviation.

Several customizable color schema are available.

The user can set the max level of the visualized expression data. This absolute value will define the upper and lower threshold of the visualized expression interval.

The filtering and the normalization can be applied to the entire data set or the selected genes only. For a general exploration, is recommended to create an expression matrix from the entire dataset. This process is done once and can be used to investigate e.g. a large network, as long there is enough memory. The other option, the extraction and visualization of selected genes only is less memory consuming. In this case, the extraction, normalization of the data has to be done again if other genes are of interest.

Example of normalized expression (Fig 10) using normal colon mucosa data. All selected experiments and all spots corresponding to a gene will be displayed as a node label.

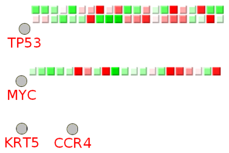

Figure 10: *Normalised expression for test genes*

Spots with expression > 3 for at least 12 out of 22 samples were kept. Maximum expression level visualized was 1.5. Expression label is missing for low expressed genes.

Improvements of the expression data upload available starting with CluePedia v1.7

Files containing one or several columns with identifiers can be imported. The order of identifier columns in the file is not important. Important is that one of those columns contains unique identifiers (no duplicate ids).

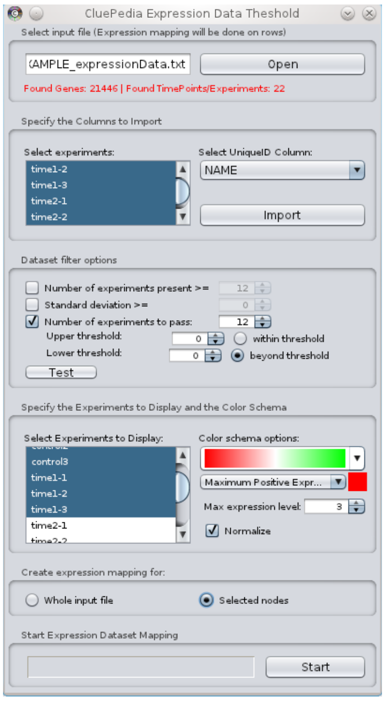

Figure 11: *CluePedia (v1.7+) expression data filters*

The column names should be unique as well. All columns containing numerical data will be considered as experiments. When analyzing the entire dataset, it is important to exclude numerical identifiers e.g. entrezGeneIDs.

The user has to choose the column with unique identifiers (in the example, the column ”NAME”) that will be used to map and select markers. Further, the user can select, analyse and visualize all expression data or just a part of it. Like this, it is possible to investigate from a large dataset one or several experimental time points of interest.

Expression data from zip archives can be uploaded starting with CluePedia v1.7.1 (Fig 12). For example, GEO expression matrix downloaded as zip archive can be uploaded in CluePedia and directly used for correlation calculation or expression data visualization. CluePedia selects automatically the data matrix. Commentary lines (marked with ”!”) are excluded.

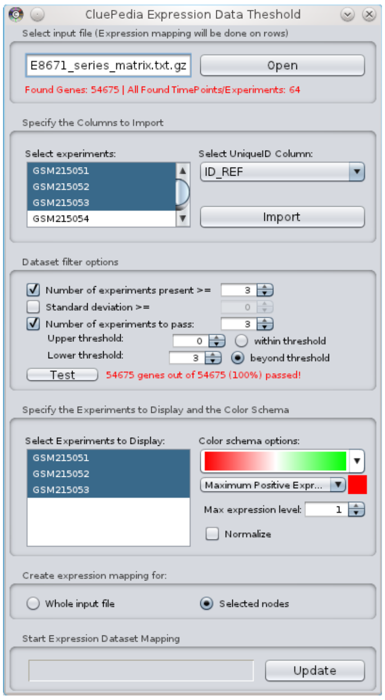

Figure 12: *CluePedia v1.7.1: expression data uploaded from zip archive*

Close expression dataset

If not needed anymore, the expression dataset should be closed. Closing the file increases the free Cytoscape memory.

Expression data extraction

The expression corresponding to selected genes can be extracted from an input file (Fig 13). The info will be saved in the output file. If the input file contains several spots for a gene, all will be extracted. The number of found and not found genes is displayed in a dialog.

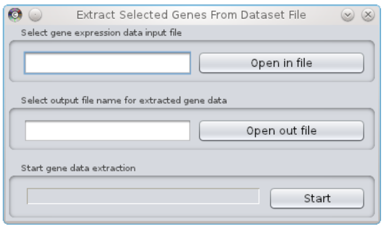

Figure 13: *Expression extraction*

Save all selected genes

Information about selected genes can be saved. Beside the symbol, entrezGeneID and synonyms, the file contains also the origin of the gene, if it was included in the initial gene list or added/enriched afterwards. The functions in which each gene is involved are mentioned. This involves a ClueGO functional analysis.

NAME	EntrezGeneID	Aliases	Origin	Functions
CEACAM7	1087	[CEA, CGM2]	Enriched Gene	
DCN	1634	[CSCD, DSPG2, PG40, PGII, PGS2, SLRR1B]	Enriched Gene	
HLA-B	3106	[AS, HLAB, MGC111087, SPDA1]	Enriched Gene	
KRT5	3852	[CK5, DDD, EBS2, K5, KRT5A]	Initial Gene	
MYC	4609	[MRTL, bHLHe39, c-Myc]	Initial Gene	
TP53	7157	[FLJ92943, LFS1, P53, TRP53]	Initial Gene	

Figure 14: *Save Gene Info*

II. Gene/Interaction Enrichment Options

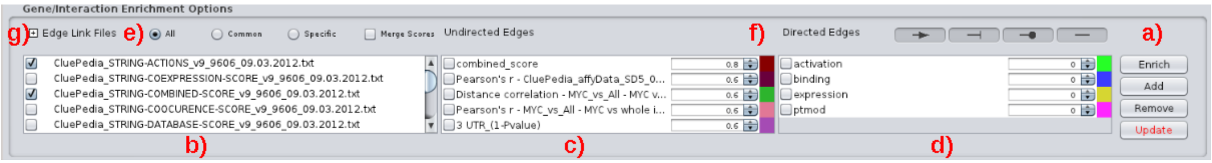

Figure 15: *Gene/interactions enrichment options*

a) Network manipulation options

- Enrich
- Add
- Remove
- Network update

b) Interaction data files

c) Edge scores

d) Edge actions

e) Undirected edge filters

f) Directed edge filters

g) Advanced options

Enrich (Find new associations for an initial set of markers)

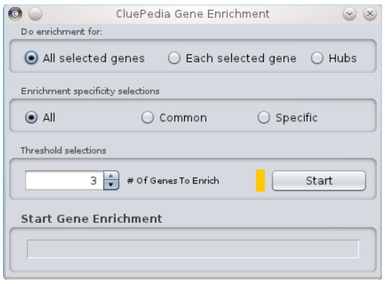

Figure 16: *Enrichment dialog*

Includes in the network genes close related to the selected gene/genes. The user selects the file/files with interactions based on which the enrichment is made. The number of enriched genes and their color can be set by the user. The enrichment dialog can be opened using the right mouse button.

If the enrichment is made for multiple genes, a data store containing all possible interactions of those genes is created. Interaction scores are extracted from all source files selected. The enrichment can be done for all or for each of the initial genes. In the first case, after sorting all interaction scores, the top ones are included in the network. Several criteria (All, Common, Specific) can be used to refine the selection (Fig 16). Similarly, interaction scores corresponding to each gene are sorted to select top related genes. The initial network will include different enriched genes, depending on the enrichment options selected. A comparison of the possible enrichment results can be seen in the Fig 17.

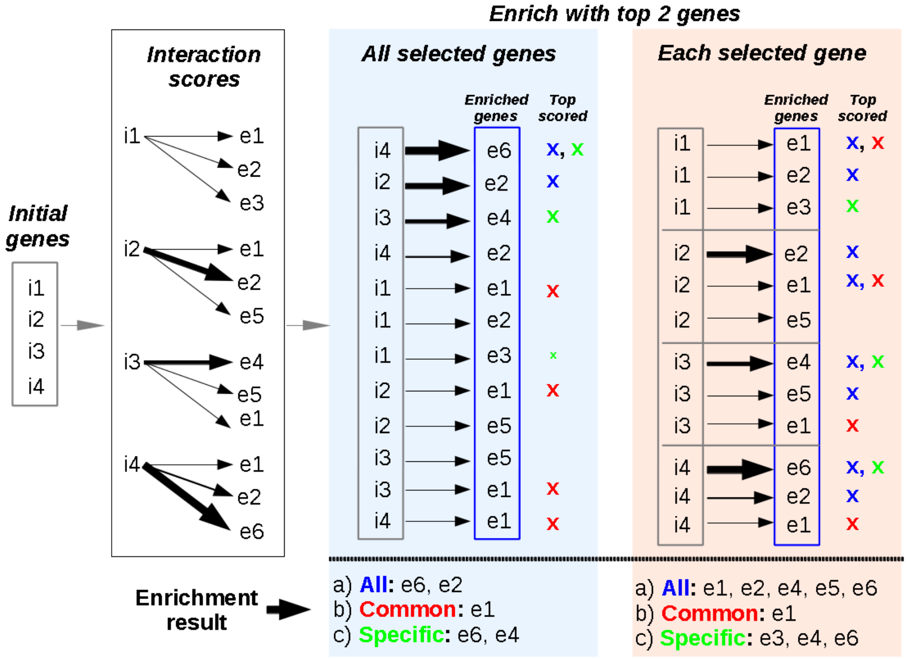

Figure 17: *Enrichment examples*

If the enrichment is made in a network of terms and genes, the enriched genes that are known to be involved in one of the functions will be automatically linked to it and displayed in the same color.

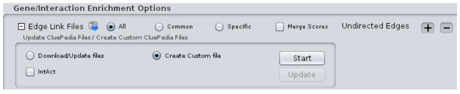

Figure 18: *Positive/negative edge score selection*

Improvements of the enrichment starting with CluePedia v1.7

Highly connected markers (hubs) with selected genes can be added to the network/pathway using the enrichment function (Fig 16). Positive and negative interaction scores can be selected and visualized on the network (Fig 18).

Add (Include a new set of markers to the current selection)

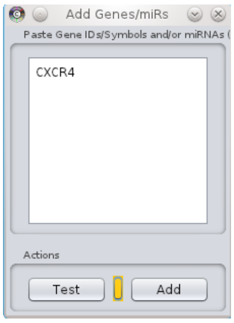

Figure 19: *Add*

Allows to include genes/miRNAs of interest in the network (Fig 19). The user can test if the IDs are found in the ID index file, and then add them on the network. The color of nodes representing added genes can be set by the user.

Remove (Discard enriched/added markers)

Selected genes/miRNAs (markers from initial list as well as added and enriched markers) or enriched genes/miRNAs (enriched and added markers only) can be removed from the network (Fig 20).

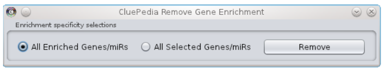

Figure 20: *Remove*

Network update (Refresh the network view with selected options)

Applies on the network the latest settings of the user.

Interactions

Markers can be linked using *in silico* or/and experimental derived information. CluePedia comes with known human and mouse interaction data. The user can create his own interaction files using experimental data (see *Edge files, advanced options*).

Known interactions

Files with known interaction are based on publicly available data from STRING, miRBase and miRRecords and other resources (*Ontologies and in silico data sources* section). Separate files are created for miRNA binding site predictions, validated binding sites as well as for each type of STRING described associations: actions, coexpression, combined score, coocurence, database score, experimental, fusion, neighborhood, textmining. The name of each file contains the type of the association, the version of the database used, the organism and the date when the file was compiled. Files can be used independently or simultaneously (depending on the free Cytoscape memory).

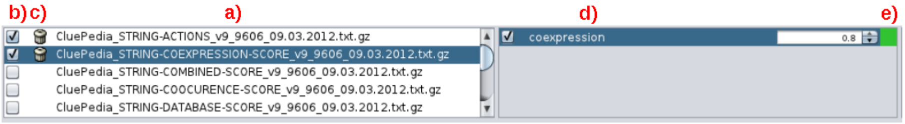

Figure 21: *Interaction files and scores*

Precompiled files available are listed (Fig 21a). Each file is loaded after selection (Fig 21b). If not needed anymore, the file should be closed (for more Cytoscape free memory) (Fig 21c). The file can be deleted using right mouse button. If a provided file is deleted (by mistake), it will be restored at the next run of the plugin.

Interactions from custom data

Experimental data (affymetrix, tissue microarray, FACS etc) can be used to visualize gene-gene/protein-protein interactions. For data format see *Data format*.
The interactions can be calculated using the ”Create custom file” feature (Fig 22).

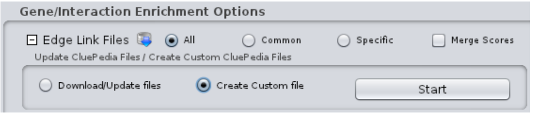

Figure 22: *Create file with custom interactions*

After importing the data, the user can filter it, as explained in *Expression data visualization*. Futher, several statistical tests can be applied to calculated interrelations for the entire file or for selected genes only (Fig 23).

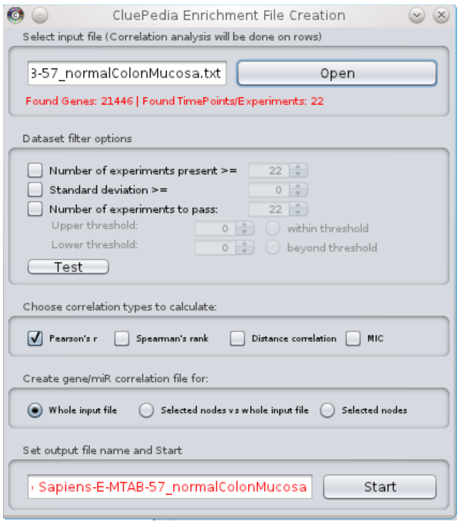

Figure 23: *Create custom file with interaction data*

Improvements of the data filtering starting with CluePedia v1.7

The user has to choose the column with unique identifiers (in the example, the column "NAME") that will be used to map and select markers.

Further, the user can import and analyse all expression data or just a part of it. Like this, it is possible to investigate from a large dataset one or several experimental time points of interest.

New filtering options are available, to avoit long calculation time and the creation of large files with results.

The user can set a threshold of the calculated correlation value to be saved in the files. Correlations with an absolut value smaller than the set threshold are filtered out.

The interrelation is filtered out if:

a) one of the correlation values calculated using different statistical tests is smaller than the

threshold (most stringent filtering out)

b) all correlations are smaller than the threshold

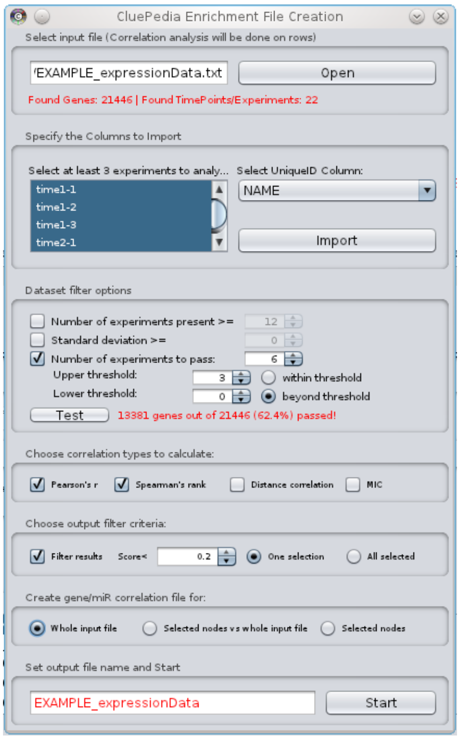

Figure 24: *Create custom file with interaction data, CluePedia v1.7+*

Expression data from zip archives can be uploaded starting with CluePedia v1.7.1 (Fig 25). For example, GEO expression matrix downloaded as zip archive can be uploaded in CluePedia and directly used for correlation calculation or expression data visualization. CluePedia selects automatically the data matrix. Commentary lines (marked with ”!”) are excluded.

Implemented statistical tests

Four correlation methods are implemented in CluePedia:

- Pearson correlation
- Spearman’s rank
- Distance correlation (DCOR) [2]

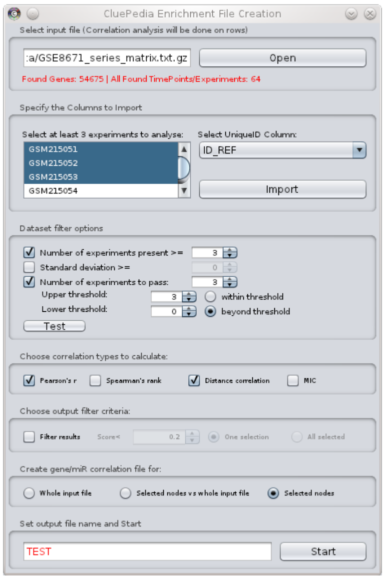

Figure 25: *CluePedia v1.7.1: expression data uploaded from zip archive*

- Maximal information criteria (MIC) [3]. Using this method, four interrelation scores are calculated:
  - MIC: reflects relationship strength
  - MAS: captures departure from monotonicity
  - MEV: shows closeness to being a function
  - MCN: captures complexity

Pearson and Spearman's rank, measures of linear dependency between two variables, can be applied for normal and not normal distributed data, respectively. DCOR and MIC reveal non linear relationships.

The correlation analysis can be done simultaneously with all methods or with a selected one, to avoid too long calculation time (MIC is particularly time and memory demanding).

Three levels of correlation analysis are possible:

- Whole input file. Correlation between all the genes from the custom file.
- Selected nodes vs all input file. Correlation between the selected node/nodes and the other genes from the dataset.

- Selected nodes. Correlation between the selected nodes only.

The correlation level selection depends on the analysis type. If the user wants to have an overview of the data, the first choice is the best. Because it is time and memory consuming, this option should be applied for a small dataset. In case of large datasets, the analysis can take very long, so the correlation of selected nodes vs all dataset is recommended. This second option is suitable for finding new genes that have a similar behavior/function with known genes. The last option shows the degree of correlation of selected genes e.g. known to be involved in a pathway.

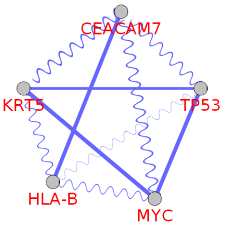

Figure 26: *Custom data, positive and negative correlation values*

The time needed for correlation depends on the method and level selected, the number of the genes and the computer power. A free memory bar helps to estimate the waiting time.

The resulting file is saved on the hard disk and included in CluePedia as an additional resource useful for further analysis.

Correlation scores are shown on the network as edges. Negative correlation values are represented as sinusoidal lines, for a better visibility (Fig 26). The user can visualize one or several correlation scores.

Interactions visualization (edges)

Edge scores

Once an interaction file is selected, the score type contained is displayed (Fig 21d). A threshold is automatically set to display the top interactions. This threshold as well as the color of the edge are customizable (Fig 21e).

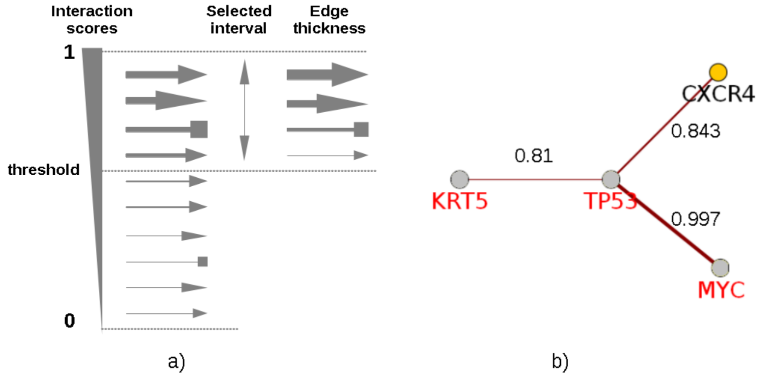

Figure 27: *Edge thickness*

The edge thickness is scaled between max and min scores found among the set criteria. The smallest score will be displayed as thinnest line (Fig 27).

Edge actions

STRING action file contains: activation, binding, expression, post translational modification (ptmod). Each type of interaction is shown with a different color. The color and the threshold are customizable.

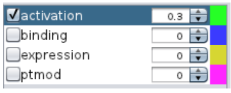

Figure 28: *Action types*

Undirected edge filters

If several interaction types are selected, the user can display all, common or specific interactions (Figures 30, 31):

- All. Displays all the edges found between genes under set criteria.
- Common. Displays interactions found in all selected files.
- Specific. Interactions found only in one file.

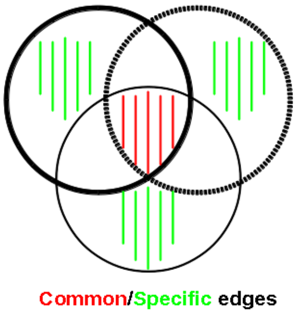

Figure 29: *Interaction information overlap*

- Merged. Combines all scores between two genes into a new, summarizing score calculated based on the value of each of the interactions. The smallest merged score will be the thinnest.

  $TotalScore = 1 - \prod_{i=0}^{n}(1 - Score_i)$

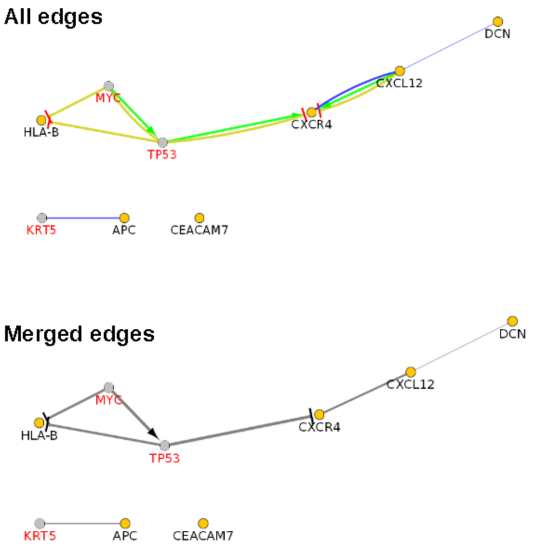

Figure 30: *Undirected edges: All vs Merged*

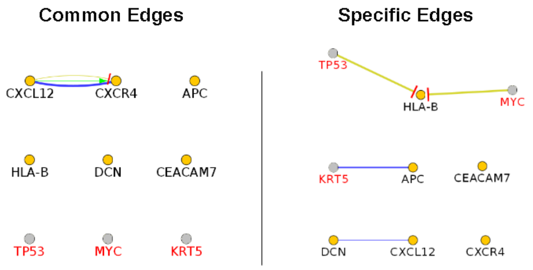

Figure 31: *Undirected edges: Common vs Specific*

Directed edge filters

All types of action are selected by default (Fig 32a). The user can choose the type of directed edge to display; e.g only activation (Fig 32b).

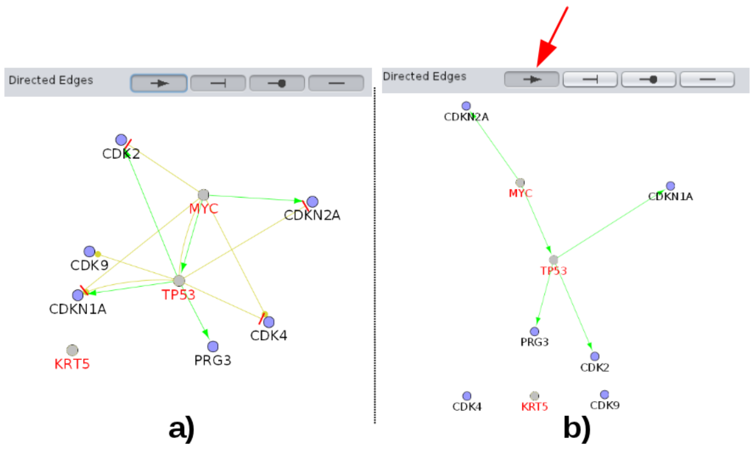

Figure 32: *Directed edge filters*

Download interactions for other organisms/Update interactions

The CluePedia plugin stores human and mouse interaction data. Precompiled files for other organisms can be downloaded by the user (Fig 33). Files corresponding to the selected organism will be proposed for download.

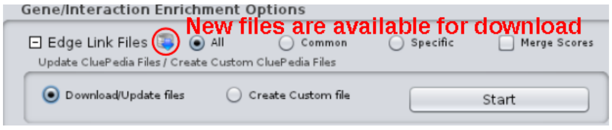

Figure 33: *Interaction data, advanced settings*

New interaction files will be ready to download for each organism following new releases of the databases used as source (Fig 34). The newly downloaded files will be added to the other CluePedia resources. If there are no new files on the server, the user is informed. A deleted CluePedia file can be uploaded again.

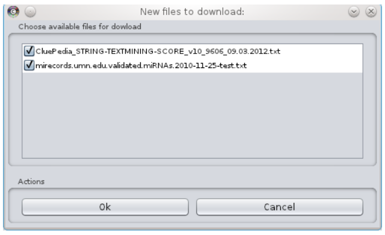

Figure 34: *Interaction data, advanced settings: update files*

ClueGO+CluePedia functionality

CluePedia can be used in combination with ClueGO. In detail analysis of terms/pathways can be done by creating nested networks corresponding to each of the pathways or by extracting from the pathways genes related to the mapped genes.

Expand pathways in nested networks

This feature provides insights into pathways. All the genes included in the pathway will be visualized in a nested network. Known and calculated interaction scores between those genes

can be compared.

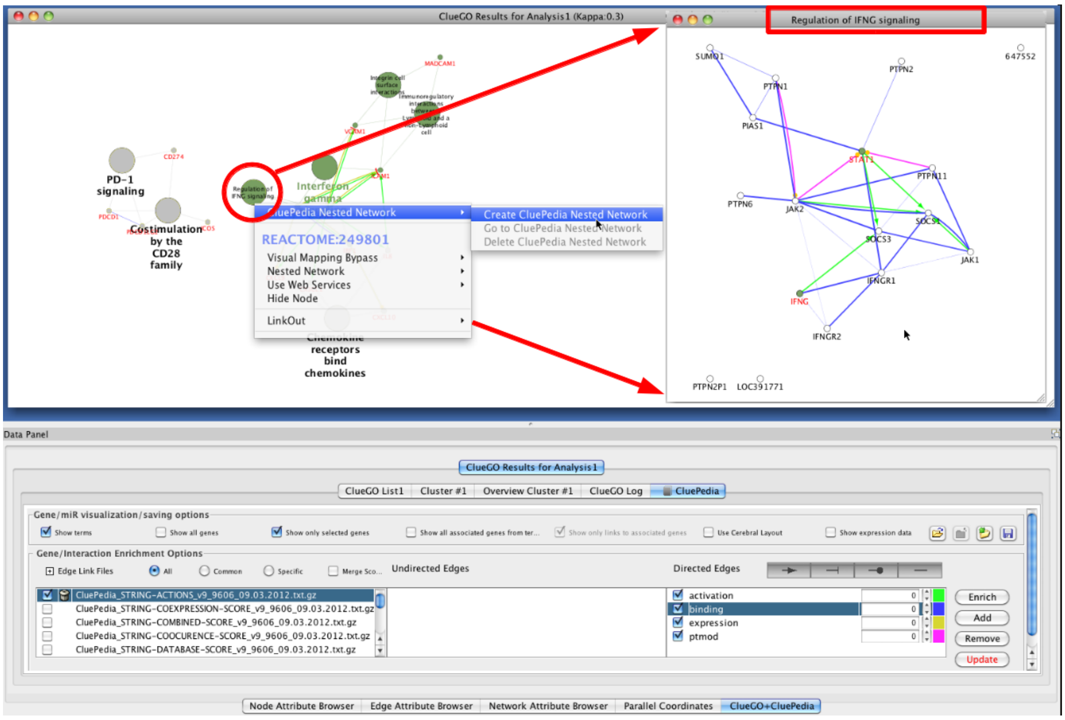

Figure 35: *CluePedia: create nested networks*

- Right mouse click on the pathway of interest, Create CluePedia Nested Network => a nested network with all associated genes (Fig 35)
  - Initial genes mapped share the color of the term. The rest of the genes included in the pathway are displayed in white.
  - The interaction scores used in the initial network will be automatically visualized in the subnetwork. Genes not passing interaction score thresholds are not linked.
  - The terms expanded in a nested network will display a small network drawn on their surface.
  - The focus on the nested network can be set by selecting the term (Right mouse click, Go to CluePedia Nested Network)
  - If the subnetwork is not needed anymore, it should be deleted.

Extract from pathways genes interracting with genes from initial list

After performing a ClueGO functional analysis, genes associated with terms can be visualized together on the network.

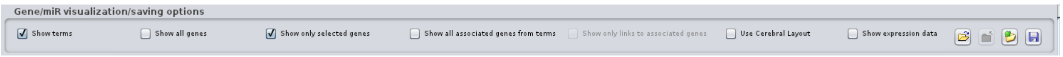

Figure 36: *ClueGO+CluePedia: visualization options*

Several options for gene visualization are available (Fig 36):

- Show all genes (Fig 37).
- Show only selected genes (Fig 38).
- Show all associated genes from terms (Fig 39).
- Show only links to associated genes (Fig 40).

Show only selected genes

Displays genes from the initial list found under the used ClueGO settings. The name of the gene is colored in red. Terms and their genes share the color. If a gene is found in two or more terms, it will have two or more colors. Terms are interconnected using kappa score, while genes based on activation scores.

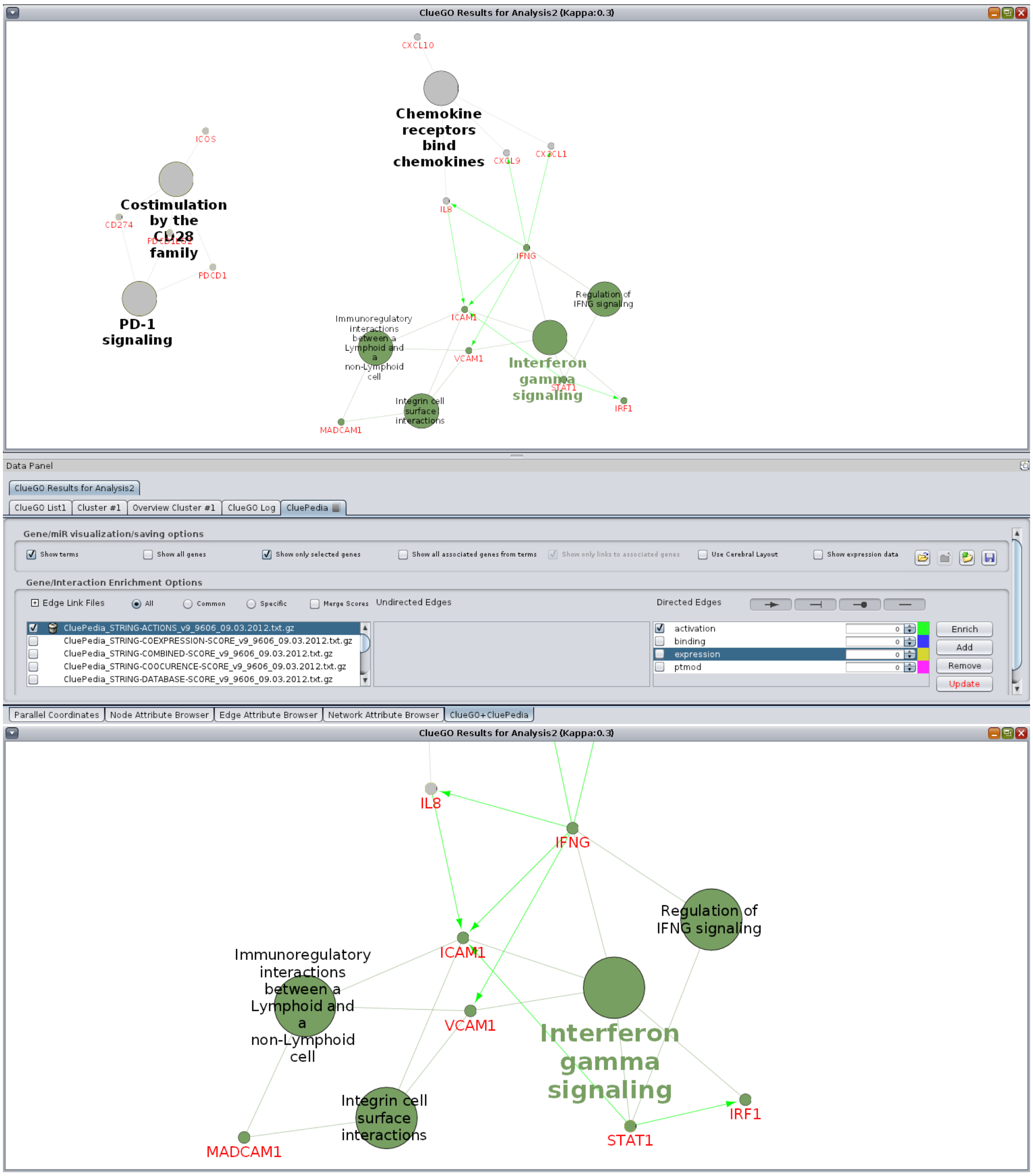

Figure 37: *ClueGO+CluePedia: functional terms and initial genes found*

Show all genes

Displays all genes included in each pathway/term. The names of the initial genes found are displayed in red. For the rest of the genes, the name is written in black.

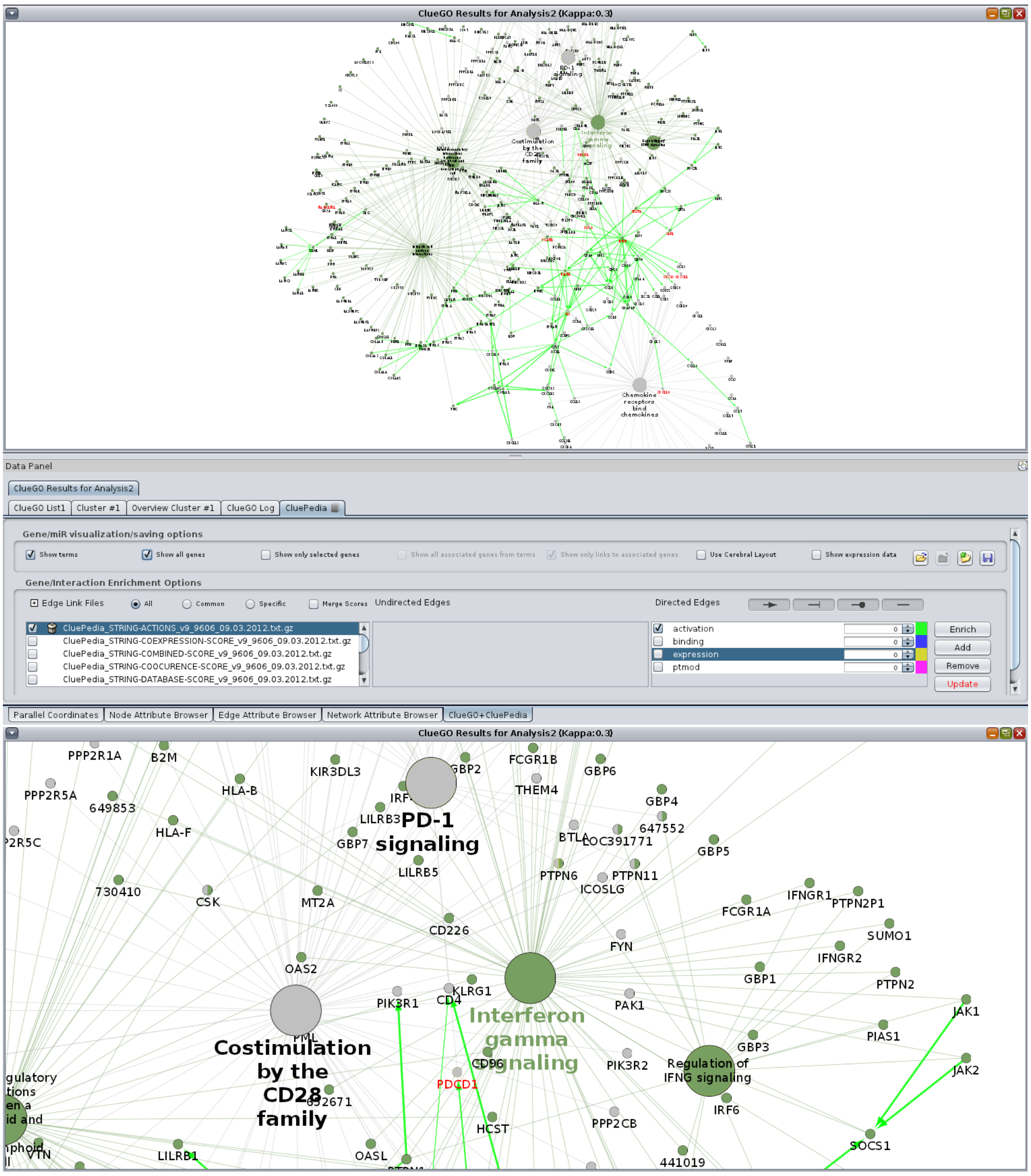

Figure 38: *ClueGO+CluePedia: Show all genes*

Show all associated genes from terms

This features applies if an interaction file is selected and scores are visualized on the network. It is used to extract from the pathway nodes and visualize genes that have an interaction link (known/calculated score) to the initial genes. The example figure was created using activation score. Interconnections between those new genes are visualized as well.

E.g. ICAM1 is linked both, to CD40LG and CD40. CD40LG is linked to CD40.

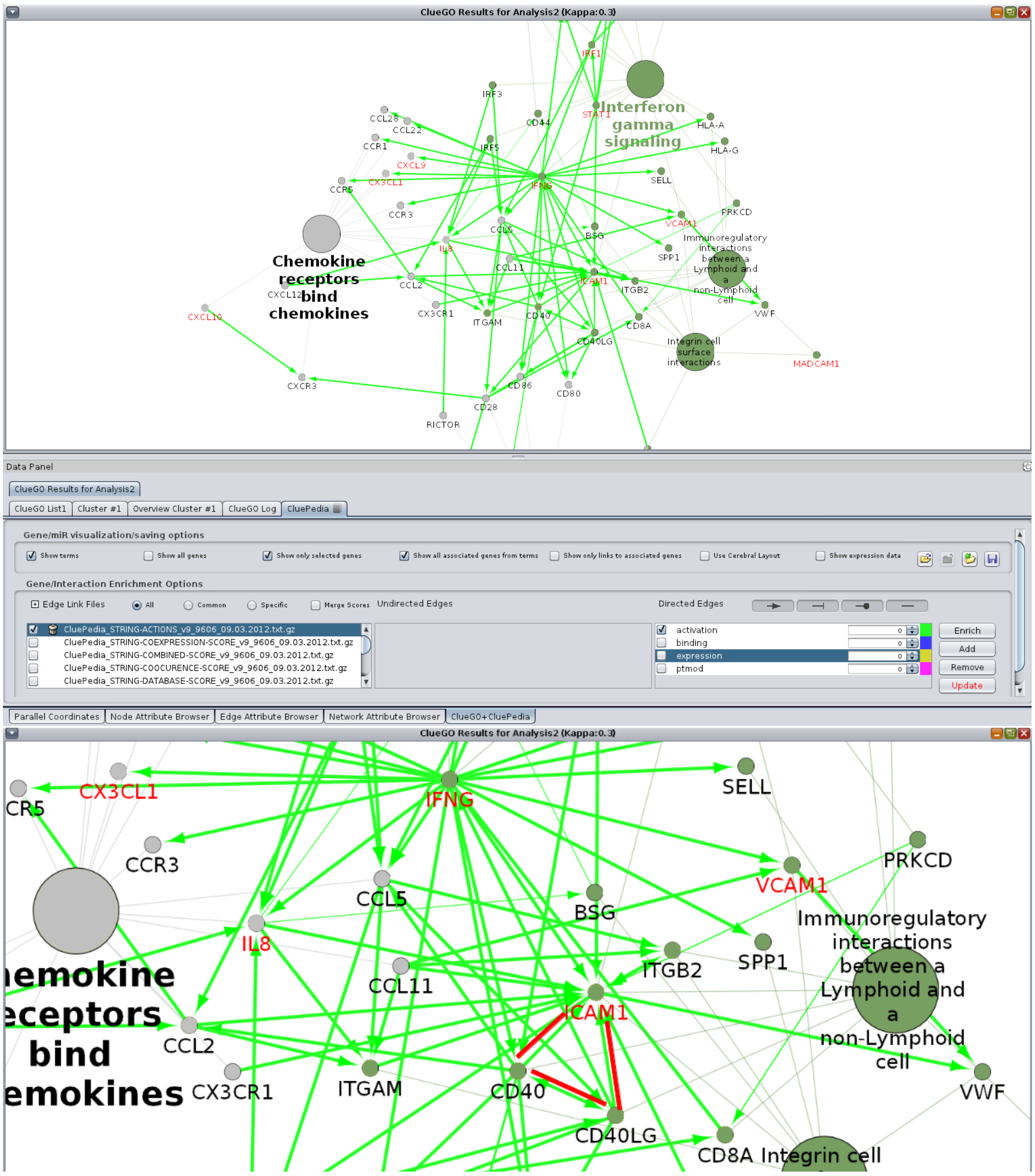

Figure 39: *ClueGO+CluePedia: functional terms and all associated genes from terms*

Show only links to associated genes

This feature simplifies the interrelation view in regard to the initial genes compared to Show all associated genes from terms. Suppresses the links between newly extracted genes from pathway nodes. Only links between initial genes and the new genes are kept. E.g. ICAM1 is linked both, to CD40LG and CD40. The link between CD40LG and CD40 is not shown.

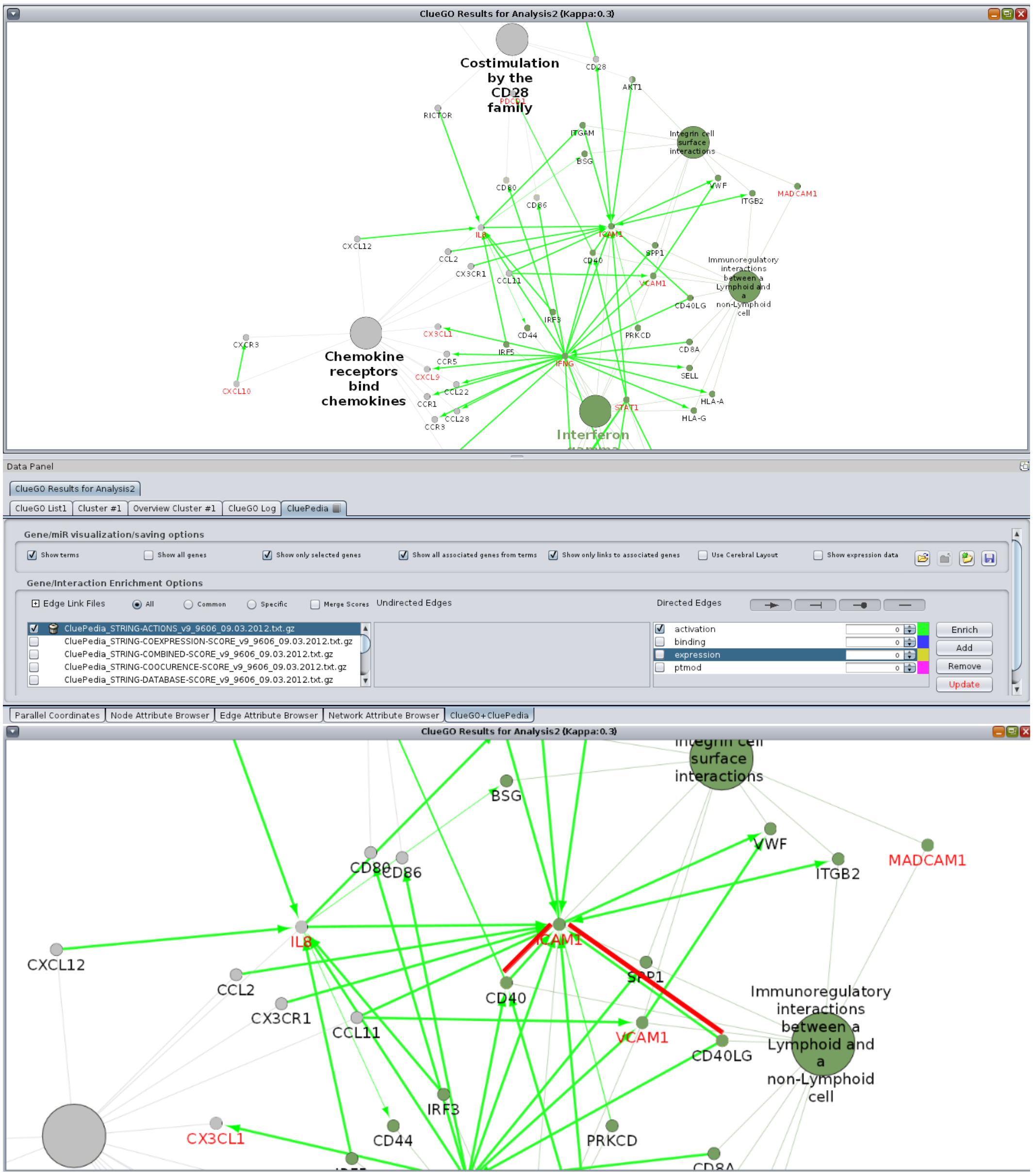

Figure 40: *ClueGO+CluePedia: functional terms, links to initial genes only*

How to...

To run those examples CluePedia and ClueGO plugins have to be saved in the .plugin folder of Cytoscape. ClueGO+CluePedia can be found under the Cytoscape Plugins menu.

Visualize known interactions

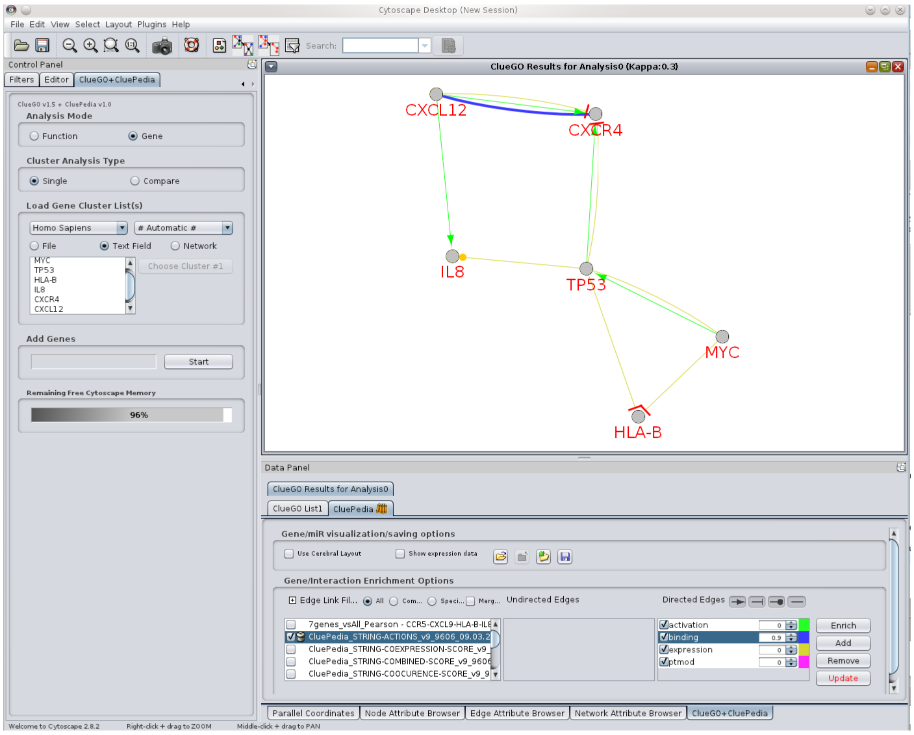

Figure 41: *Visualize known interactions*

- Select Analysis Mode: Gene
- Keep Standard Settings:
  - Cluster Analysis Type: Single
  - Organism: Homo Sapiens
  - ID type: Automatic
- Gene Cluster source: Text Field

- Copy/type genes: MYC, TP53, HLA-B, IL8, CXCR4, CXCL12.
- Start the analysis => Genes are mapped as nodes of the network. A table with gene synonyms is created.
- Select CluePedia panel
- *Optional* Select: Use Cerebral layout to see chromosomal location of the genes
- Select the interaction source file: CluePedia STRING ACTIONS
- Select Action types to visualize: all actions, all scores
- Update network => Known action scores (activation, binding, expression) between genes are visualized as edges of the network. Edges are scored between the min and max scores found. The smallest score is the thinnest.
- Set the binding score to 0.9.
- Update network => Only the binding score > 0.9 is visualized on the network.

Calculate interactions between markers using experimental data

- Select Analysis mode Gene/miRNAs
- Paste genes in Text field
- Click Start
- Select genes from the network
- Expand Edge Link Files
- Select Create Custom File
- Click Start

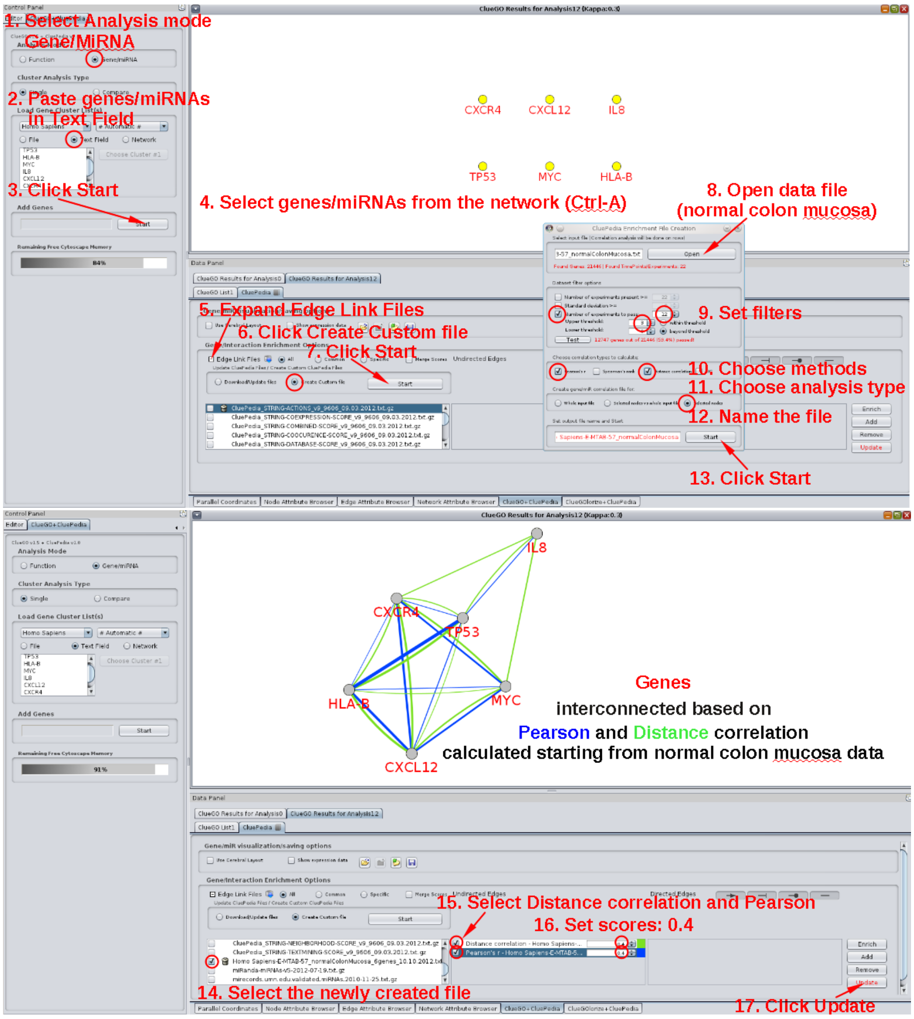

Figure 42: *Visualize known interactions*

- Open experimental data file containing normal colon mucosa. For custom files, see File format description.
- Set filters (Filters are described in Expression data visualization section)
- Select Pearson and Distance correlation methods
- Choose the type of analysis: Selected Nodes only
- Set the name of the new file
- Start calculation. => The new file with calculated correlation is added as resource to

CluePedia.

- Select edges to visualize (Pearson and Distance correlation)
- Set scores: 0.4
- Click Update => Calculated interrelations between genes will be visualized.

Make gene/miRNA enrichment

New markers, related to the genes/miRNAs from the network can be extracted from interaction files using the enrichment function of CluePedia. The enrichment can be done based on one (recommended) or several interaction scores.

From source files are initially extracted all scores corresponding to selected genes/miRNAs, sorted based on their value and then the top ones are visualized. Scores having the same value are alphabetically sorted and only the number of the markers set by the user is shown. Because of this, is possible that some interactions passing the score value threshold are not visualized. An enrichment with a higher number of markers could bring all interactions above the set threshold in the network. Validated miRNAs have the score 1.

Find new related genes

- Upload genes of interest in CluePedia
- Select CluePedia STRING EXPERIMENTAL file
- Select expression score
- Set score value: 0.8
- Select genes from the network
- Click Enrich. => Enrichment dialog

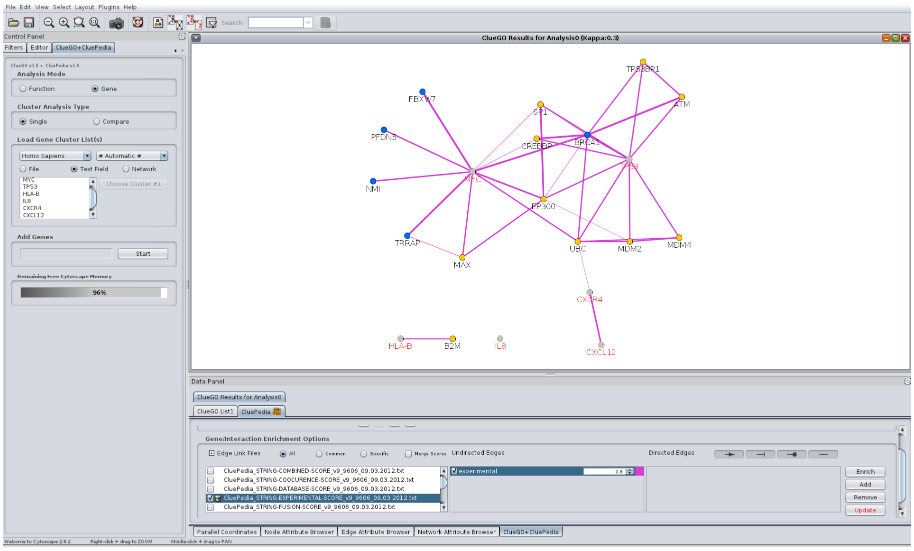

Figure 43: *Find new related genes*

- *Keep predefined settings (All Selected genes, All, 10 genes, yellow color).* => top 10 scored genes related to all selected genes.
- Select MYC
- Enrich. Set the number of the new genes to 5 and blue color for the nodes. => next 5 top scored genes related to MYC are added to the network.

Find miRNAs predicted/validated to bind genes of interest

- Upload genes of interest in CluePedia
- Select CluePedia miRanda miRNAs file and miRanda SCORE (0.6)
- Select genes from the network
- Select CluePedia mirecords.umn.edu miRNAs file and validated miRNA score (all, 0)
- Update. Shows which of the miRanda predicted interactions (green edges) were validated (brown edges). A single interaction from the predicted ones was validated. To find other validated miRNA/gene interactions:

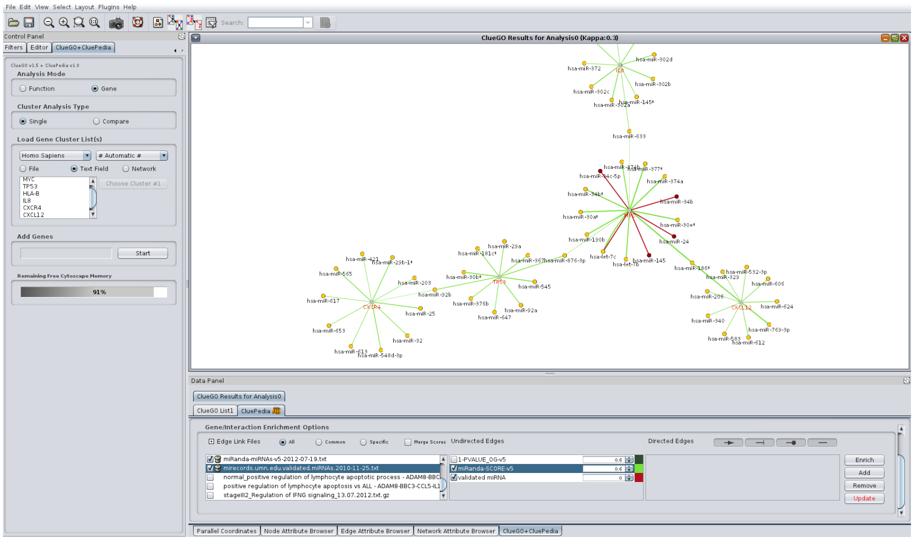

Figure 44: *Find predicted/validated miRNAs*

- Unselect miRanda SCORE
- Set validated miRNA score to 0
- Select MYC
- Enrich. Set: 5 miRNAs, node color brown. => top 5 scored miRNAs binding to MYC are added.
- Select miRanda SCORE
- Update

Find functions that could be affected by miRNAs

- Select Analysis mode Gene/miRNAs
- Paste miRNAs in CluePedia
  (e.g. hsa-miR-20a, hsa-miR-31, hsa-miR-143, hsa-miR-145)
- Click Start

- Select all 4 miRNAs from the network
- Select CluePedia miranda miRNAs file and miRanda score (0.6)

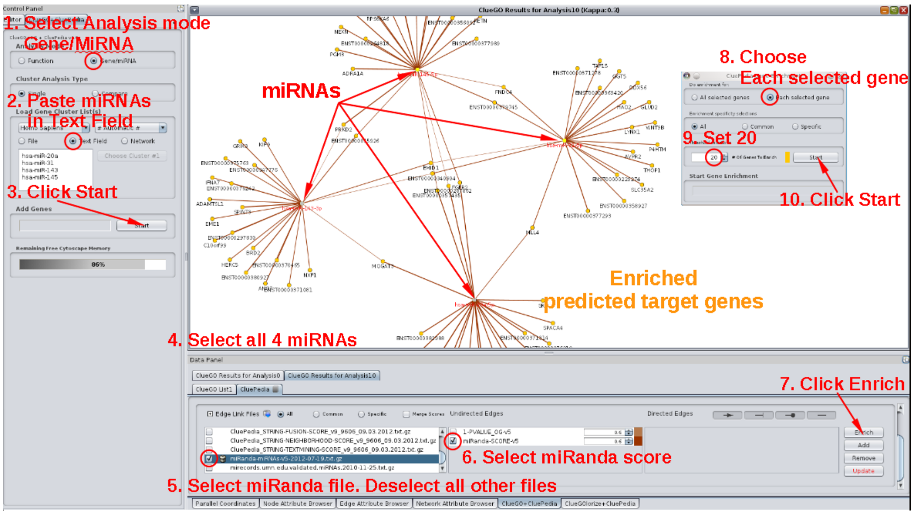

Figure 45: *Target genes of hsa-miR-20a, hsa-miR-31, hsa-miR-143, hsa-miR-145*

- Click Enrich => Enrichment dialog
- Select Each selected gene/miRNAs,
- Set 20 genes. *Keep the other standard settings.*
- Click Start => top 20 predicted target genes corresponding to each miRNA.
- Select all miRNAs and genes (Ctrl-A)
- Click Functions (ClueGO+CluePedia panel)
- Click Network (Get genes from network)
- Click Load Attributes
- Select GOID0 (*Check in the Node attribute panel the name of the column containing the ids*)
- Choose Cluster (Upload the selected genes/miRNAs)

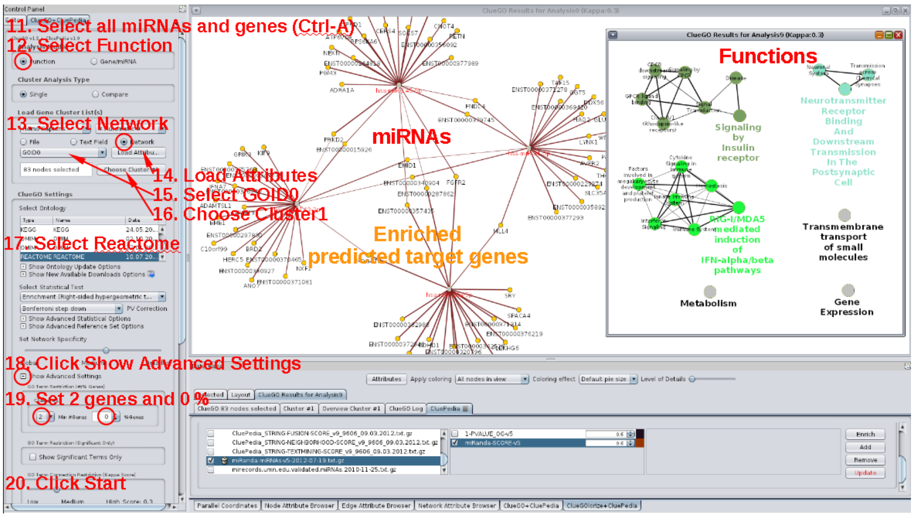

Figure 46: *Network showing the biological role of target genes of hsa-miR-20a, hsa-miR-31, hsa-miR-143, hsa-miR-145*

- Select Reactome
- Click Show Advanced Settings
- Set 2 gene/term.
- Set 0 perc/term. *Keep all the other standard settings*
- Click Start

  => Network with functions associated with target genes.

Enrich a network showing two lists of genes/miRNAs

Enrichments can be done on a network comparing two lists of genes/miRNAs. This kind of network displays nodes colored in the selected gradient (e.g. red-white-green).

The new genes showed as white nodes, will have a colored border corresponding to the enrichment color.

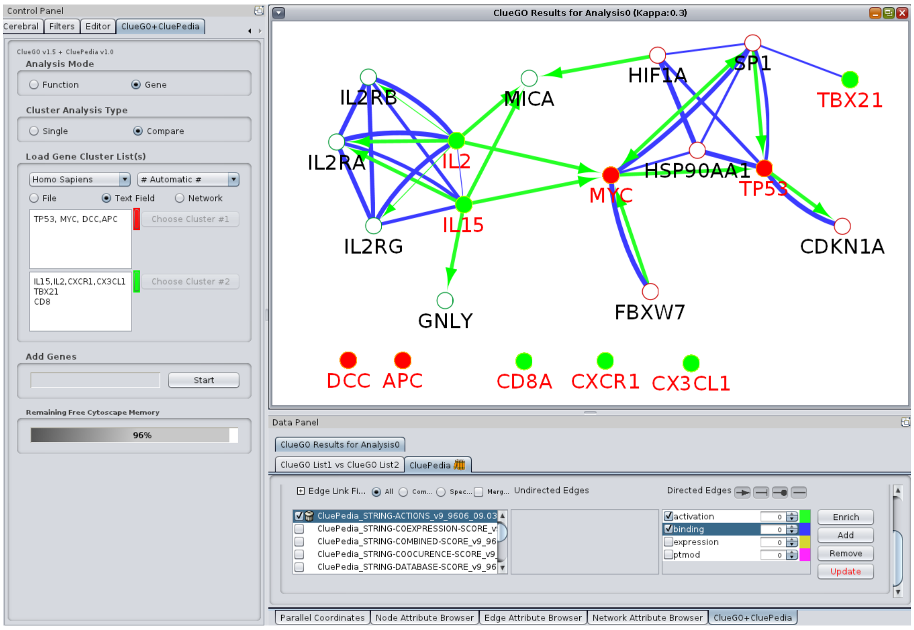

Figure 47: *Network showing enrichment for two lists of genes (red, green)*

Switch the network and pathway-like view

Click the ”Use Cerebral layout” selection box. The genes from the network (Fig 48a) will automatically displayed in cellular compartments (Fig 48b). Note: the Cerebral layout allows a single edge type betweent the nodes.

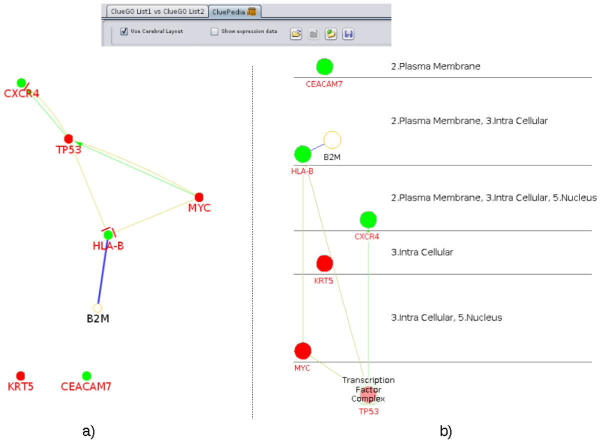

Figure 48: *Switch between the network and pathway-like view*

Upload new available example and ontology files or new organisms

Under analysis mode ”Functions”, click the ”Show New Available Download Options”(Fig 49).

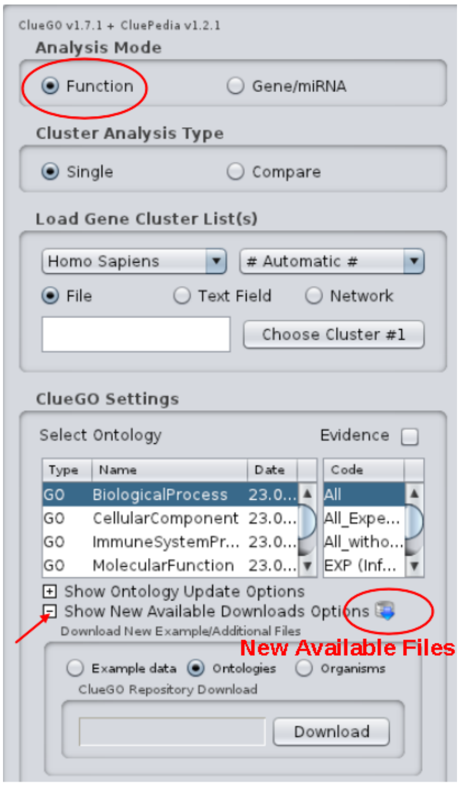

Figure 49: *Upload new available example and ontology files or new organisms*

See node and edge attributes

See node/edge attributes: Select nodes/edges of interest, select Cytoscape Node/Edge Attribute Browser, Select All Attributes

Data format

Data file specification: first two columns should contain IDs, the rest is data. Each column should have unique name. Any kind of data can be visualized.

Data file format starting with CluePedia v1.7

IDs:
Column 1 and 2

Data

NAME	Symbol	cancerLine_1	cancerLine_2	cancerLine_3	normal_1	normal_2	normal_3
206328_at	CDH15	2.26	2.24	2.30	2.30	2.30	2.29
206327_s_at	CDH15	2.34	2.29	2.57	2.29	2.33	2.31
206517_at	CDH16	1.84	1.80	1.82	1.79	1.86	1.93
209847_at	CDH17	2.27	2.29	2.27	10.87	11.12	7.67
206280_at	CDH18	2.42	2.40	2.51	2.40	2.41	2.47
206898_at	CDH19	3.47	3.31	3.73	4.04	4.15	4.14
203440_at	CDH2	2.91	2.81	2.86	2.79	2.88	4.11

Figure 50: *Data file format*

Files containing one or several columns with identifiers can be uploaded. The order of identifier columns in the file is not important. Important is that one of those columns has to contain unique identifiers (no duplicate ids).
The column names should be unique as well. All columns containing numerical data will be considered as experiments. When analyzing the entire dataset, it is important to exclude numerical identifiers e.g. entrezGeneIDs.

Symbol	UniqueID	otherID1	otherID2	control1	control2	time1-1	time1-2	time2-1	time2-2	time3-1	time3-2
15E1.2	214711_at	otherID1-1	otherID2-1	4.32	5.25	5.44	6.20	4.18	5.58	6.41	5.07
2'-PDE	214826_at	otherID1-2	otherID2-2	4.55	4.86	5.71	4.55	4.12	4.37	6.65	5.06
3.8-1	214186_s_at	otherID1-3	otherID2-1	4.53	4.59	4.99	4.81	4.22	4.54	7.60	4.92
76P	211337_s_at	otherID1-4	otherID20	2.75	2.96	2.77	2.79	3.02	2.87	4.47	2.85
76P	213266_at	otherID1-5	otherID21	2.56	2.64	2.81	2.91	3.47	2.06	2.83	2.59
A2BP1	221217_s_at	otherID1-6	otherID22	2.21	2.26	2.37	2.32	2.16	2.22	2.62	2.23
A2M	217757_at	otherID1-7	otherID23	10.73	10.25	10.77	9.11	11.84	9.18	7.09	9.55
A4GALT	219488_at	otherID1-8	otherID24	2.91	2.96	2.99	2.92	2.81	3.59	3.08	2.88

Figure 51: *Data file format starting with CluePedia v1.7*

Publicly available expression data

Gene Expression Omnibus (GEO, [12]) and ArrayExpress ([13]) are repositories for large scale experiments. Raw or normalized expression data, as well as supporting information like experiment description or sample annotation can be downloaded. For data interpretation is very important to understand the experimental conditions used.

Public available data from GEO and ArrayExpress can be uploaded in ClueGO as data matrix or after the integration of annotations, in concordance with CluePedia format.

CluePedia example is based on publicly available microarray data downloaded from ArrayExpress: accession numbers:

E-MTAB-863, Link: http://www.ebi.ac.uk/arrayexpress/experiments/E-MTAB-863

E-MTAB-57, Link: http://www.ebi.ac.uk/arrayexpress/experiments/E-MTAB-57

From those datasets 22 normal colon mucosa (E-MTAB57) and 57 tumor samples from stage III colorectal cancer patients (E-GEOD-37892) were selected. Data was formatted in CluePedia format. Those files can be downloaded using the download/update function.

Organism and ID types

The CluePedia plugin stores human and mouse interaction data. The user interested in other organisms can download automatically precompiled files from our website. The download has to be done once, and the time to download differs from organism to organism, depending on the data size. Interaction data will be updated at each new release of the sources used. Data for 20 organisms are available, and upon request other organisms can be added. The ClueGO plugin v1.5 contains 23 organisms.

CluePedia recognizes automatically gene and protein identifiers, symbols, synonyms: AccessionIDs, Affymetrix IDs, EnsProtID, EnsGene/TranscriptID, EntrezGeneID, GI, IPI, RefSeqID Protein, UniProtKB_AC, UniProtKB_ID, SymbolID, SWIS_ID. The automatical recognition of the ID types is made using the provided ID index file. This file stores entrez gene ids and symbols and allows fast analysis and synonyms detection. The ID index file can be extended with custom IDs by the user. Using the ID index file, the user query list containing a mixture of symbol types will be automatically mapped.

Update Ontologies and Interaction data

ClueGO and CluePedia allow an up to date analysis at any time.

The update functions provide an easy integration of the most recent ontology and information. The source files are automatically downloaded and based on this, new precompiled

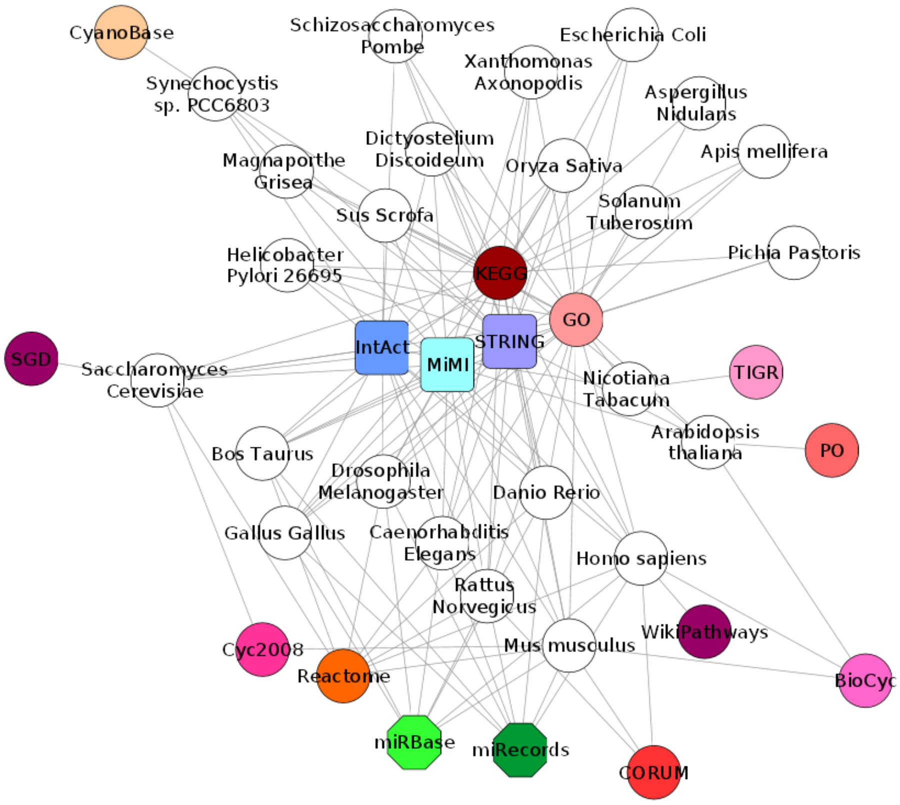

Figure 52: *CluePedia v1.7, organisms and data sources*

files are created. The new created files are added to initially provided files in the .cluegoplugin folder. If this folder is deleted or damaged, it will be restored with the initial configuration at the first run of the plugins.

At each new release of the data sources, new CluePedia precompiled files will be prepared and made available for download on our website (http://www.ici.upmc.fr). The user will be able to automatically download the files corresponding to the organism of interest.

ClueGO uses GO, KEGG and REACTOME data and other resources. The latest version of the plugin provides new ontologies: Plant Ontologies (PO), CORUM, SGD, MetaCyc data and WikiPathways. The downloaded data is precompiled in ClueGO format.

The update function of ClueGO was extended. Beside the update of GO and KEGG, is now possible to automatically update REACTOME. GOSlim and PO information is updated in the same time with GO (option mentioned in .properties file). ClueGO Documentation describes in detail the ontology and annotation sources used.

The custom GO ontologies (branches of GO) created by the user from a GO term of interest are updated in the same way as GO provided files.

An important new feature is the update of the ID Index file that will provide the latest gene information, as compiled from most recent NCBI resouses that are accessed during the update.

Ontologies and *in silico* data sources

CluePedia and ClueGO plugins contain or provide precompiled files based on the following sources:

- The Gene Ontology (GO) project [8] aims to capture the increasing knowledge on gene function in a controlled vocabulary applicable to all the organisms. GO describes gene products in terms of their associated biological processes, cellular components and molecular functions.
  Link: http://www.geneontology.org/

- Kyoto Encyclopedia of Genes and Genomes (KEGG) [9] is a database of biological systems that integrates genomic, chemical and systemic functional information.
  Link: http://www.genome.jp/kegg/
  The information from KEGG is now updated over the REST-style version API since the older SOAP API will be discontinued after Dec. 31 2012.
  (http://www.kegg.jp/kegg/rest/keggapi.html)

- REACTOME [14, 15] is an open-source, open access, manually curated and peer-reviewed pathway database.
  Link: http://www.reactome.org/
  XML Reactome data is downloaded as:
  http://www.reactome.org/download/current/ReactomeToBioSystems.zip

- STRING, database of known and predicted protein interactions, provides direct (physical) and indirect (functional) associations [4, 5]. STRING version 9.0, available at

http://string.embl.de/, covers more than 1100 organisms. Link: http://string-db.org/

- miRBase is the primary online repository for all microRNA sequences and annotation [6]. miRBase version 16, contains over 15,000 microRNA gene loci in over 140 species, and over 17,000 distinct mature microRNA sequences.
  Link: http://www.mirbase.org/

- miRecords [7] is a resource for animal miRNA-target interactions. miRecords consists of two components. The Validated Targets component is a large, high-quality database of experimentally validated miRNA targets resulting from meticulous literature curation. The Predicted Targets component of miRecords is an integration of predicted miRNA targets produced by 11 established miRNA target prediction programs.
  Link: http://mirecords.umn.edu/miRecords/

- The Plant Ontology (PO) [16] is a controlled vocabulary that describes plant anatomy and morphology and stages of development for all plants.
  Link: http://www.plantontology.org/

- The CORUM database [17] (MIPS) provides a resource of manually annotated protein complexes from mammalian organisms. Annotation includes protein complex function, localization, subunit composition, literature references and more. All information is obtained from individual experiments published in scientific articles, data from high-throughput experiments is excluded.
  Link: http://mips.helmholtz-muenchen.de/genre/proj/corum

- The Saccharomyces Genome Database (SGD) [18] provides comprehensive integrated biological information for the budding yeast Saccharomyces cerevisiae.
  Link: http://www.yeastgenome.org/

- Cyc2008 [19] provides an up-to-date reference set of yeast protein complexes for both experimental biologists who work on yeast protein complexes and computational biologists who are interested in predicting protein-protein interactions and protein complexes

in yeast.
Link: http://wodaklab.org/cyc2008/

- MetaCyc [20] is a database of nonredundant, experimentally elucidated metabolic pathways.
  Link: http://metacyc.org/

- WikiPathways [21] a public wiki for pathway curation.
  Link: http://www.wikipathways.org

- IntAct [22] provides a freely available, open source database system and analysis tools for molecular interaction data. All interactions are derived from literature curation or direct user submissions and are freely available.
  Link: http://www.ebi.ac.uk/intact/

- MiMI (Michigan Molecular Interactions) [23] provides access to the knowledge and data merged and integrated from numerous protein interactions databases.

Bibliography

[1] G Bindea, B Mlecnik, H Hackl, P Charoentong, M Tosolini, A Kirilovsky, WH Fridman, F Pages, Z Trajanoski, J Galon. ClueGO: a Cytoscape plug-in to decipher functionally grouped gene ontology and pathway annotation networks. *Bioinformatics*, 25(8):1091–3, 2009.

[2] GJ Szekely, M Rizzo. Brownian distance covariance. *Annals of Applied Statistics*, pages 1233 – 1303, 2009.

[3] DN Reshef, YA Reshef, HK Finucane, SR Grossman, G McVean, PJ Turnbaugh, ES Lander, M Mitzenmacher, PC Sabeti. Detecting novel associations in large data sets. *Science*, 334(6062):1518–24, 2011.

[4] von Mering C., Jensen L.J., Snel B., Hooper S.D., Krupp M., Foglierini M., Jouffre N., Huynen M.A., Bork P. STRING: known and predicted protein-protein associations, integrated and transferred across organisms. *Nucleic Acids Res*, 33:D433–D437, 2005.

[5] D Szklarczyk, A Franceschini, M Kuhn, M Simonovic, A Roth, P Minguez, T Doerks, M Stark, J Muller, P Bork, LJ Jensen, C von Mering. The STRING database in 2011: functional interaction networks of proteins, globally integrated and scored. *Nucleic Acids Res*, 39(Database issue):D561–8, 2011.

[6] A Kozomara, S Griffiths-Jones. miRBase: integrating microRNA annotation and deep-sequencing data. *Nucleic Acids Res*, 39(Database Issue):D152–D157, 2011.

[7] F Xiao, Z Zuo, G Cai, S Kang, X Gao, T Li. miRecords: an integrated resource for microRNA-target interactions. *Nucleic Acids Res*, 37:D105–D110, 2009.

[8] M Ashburner, C A Ball, J A Blake, D Botstein, H Butler, J M Cherry, A P Davis, K Dolinski, S S Dwight, J T Eppig, M A Harris, D P Hill, L Issel-Tarver, A Kasarskis, S Lewis, J C Matese, J E Richardson, M Ringwald, G M Rubin, G Sherlock. Gene ontology: tool for the unification of biology The Gene Ontology Consortium. *Nat Genet*, 25:25–29, 2000.

[9] M Kanehisa, S Goto, S Kawashima, A Nakaya. The KEGG databases at GenomeNet. *Nucleic Acids Res*, 30:42–46, 2002.

[10] A Barsky, JL Gardy, RE Hancock, T Munzner. Cerebral: a Cytoscape plugin for layout of and interaction with biological networks using subcellular localization annotation. *Bioinformatics*, 23(8):1040–2, 2007.

[11] P Shannon, A Markiel, O Ozier, N S Baliga, J T Wang, D Ramage, N Amin, B Schwikowski, T Ideker. Cytoscape: a software environment for integrated models of biomolecular interaction networks. *Genome Res*, 13:2498–2504, 2003.

[12] T Barrett, DB Troup, SE Wilhite, P Ledoux, C Evangelista, IF Kim, M Tomashevsky, KA Marshall, KH Phillippy, PM Sherman, RN Muertter, M Holko, O Ayanbule, A Yefanov, A Soboleva. NCBI GEO: archive for functional genomics data sets–10 years on. *Nucleic Acids Res*, 39(Database issue):D1005–10, 2011.

[13] H Parkinson, U Sarkans, N Kolesnikov, N Abeygunawardena, T Burdett, M Dylag, I Emam, A Farne, E Hastings, E Holloway, N Kurbatova, M Lukk, J Malone, R Mani, E Pilicheva, G Rustici, A Sharma, E Williams, T Adamusiak, M Brandizi, N Sklyar, A Brazma. ArrayExpress update - an archive of microarray and high-throughput sequencing-based functional genomics experiments. *Nucleic Acids Res*, 39(Database issue):D1002–4, 2011.

[14] G Wu, X Feng, L Stein. A human functional protein interaction network and its application to cancer data analysis. *Genome Biol*, 11(5):R53, 2010.

[15] D Croft, G O'Kelly, G Wu, R Haw, M Gillespie, L Matthews, M Caudy, P Garapati, G Gopinath, B Jassal, S Jupe, I Kalatskaya, S Mahajan, B May, N Ndegwa, E Schmidt, V Shamovsky, C Yung, E Birney, H Hermjakob, P D'Eustachio, L Stein. Reactome: a database of reactions, pathways and biological processes. *Nucleic Acids Res*, 39(Database Issue):D691–7, 2011.

[16] S Avraham, CW Tung, K Ilic, P Jaiswal, EA Kellogg, S McCouch, A Pujar, L Reiser, SY Rhee, MM Sachs, M Schaeffer, L Stein, P Stevens, L Vincent, F Zapata, D Ware. The Plant Ontology Database: a communityresource for plant structure and developmental stages controlledvocabulary and annotations. *Nucleic Acids Res.*, 36(Database issue):D449–54, 2008.

[17] A Ruepp, B Waegele, M Lechner, B Brauner, I Dunger-Kaltenbach, G Fobo, G Frishman, C Montrone, HW Mewes. CORUM: the comprehensive resource of mammalian protein complexes–2009. *Nucleic Acids Res.*, 38(Database issue):D497–501, 2010.

[18] JM Cherry, EL Hong, C Amundsen, R Balakrishnan, G Binkley, ET Chan, KR Christie, MC Costanzo, SS Dwight, SR Engel, DG Fisk, JE Hirschman, BC Hitz, K Karra, CJ Krieger, SR Miyasato, RS Nash, J Park, MS Skrzypek, M Simison, S Weng, ED Wong. Saccharomyces Genome Database: the genomics resource of budding yeast. *Nucleic Acids Res.*, 40(Database issue):D700–5, 2012.

[19] S Pu, J Wong, B Turner, E Cho, SJ Wodak. Up-to-date catalogues of yeast protein complexes. *Nucleic Acids Res.*, 37(3):825–31, 2009.

[20] R Caspi, T Altman, JM Dale, K Dreher, CA Fulcher, F Gilham, P Kaipa, AS Karthikeyan, A Kothari, M Krummenacker, M Latendresse, LA Mueller, S Paley, L Popescu, A Pujar, A Shearer, P Zhang, PD Karp. The MetaCyc Database of metabolic pathways and enzymes and the BioCyc collection of Pathway/Genome Databases. *Nucleic Acids Res.*, 38(1):D473–D479, 2010.

[21] T Kelder, MP van Iersel, K Hanspers, M Kutmon, BR Conklin, CT Evelo, AR Pico. WikiPathways: building research communities on biological pathways. *Nucleic Acids Res.*, 40(Database issue):D1301–7, 2012.

[22] Breuza L Bridge A Broackes-Carter F Chen C Duesbury M Dumousseau M Feuermann M Hinz U Jandrasits C Jimenez RC Khadake J Mahadevan U Masson P Pedruzzi I Pfeiffenberger E Porras P Raghunath A Roechert B Orchard S Hermjakob H. Kerrien S, Aranda B. The IntAct molecular interaction database in 2012. *Nucleic Acids Res.*, 40(Database issue):D841–6, 2012.

[23] Ade A Bookvich A Gao J Mahavisno V Wright Z Chapman A Jayapandian M Ozgur A Tian Y Cavalcoli J Mirel B Patel J Radev D Athey B States D Jagadish HV. Tarcea VG, Weymouth T. Michigan molecular interactions r2: from interacting proteins to pathways. *Nucleic Acids Res.*, 37(Database issue):D642–6, 2009.
